# Investigation of Vascular Behaviour and Tissue Response Following Intra-Arterial Injection of Poly D,L-Lactic Acid with Hyaluronic Acid Hybrid Biostimulators (Juvelook^®^ and Juvelook Volume^®^) in a Rabbit Ear Model

**DOI:** 10.3390/jfb17090447

**Published:** 2026-09-04

**Authors:** Vasanop Vachiramon, Tussapon Boonyarattanasoonthorn, Kasem Rattanapinyopituk, Jeeraprapha Duangbupha, Vudhiporn Limprasutr, Anusak Kijtawornrat, Jovian Wan

**Affiliations:** 1 Division of Dermatology, Ramathibodi Hospital, Mahidol University, Bangkok 10400, Thailand; 2 Chulalongkorn University Laboratory Animal Center (CULAC), Pratumwan, Bangkok 10330, Thailand; tussapon.b@chula.ac.th (T.B.); jeeraprapha.d@chula.ac.th (J.D.); vudhiporn.l@pharm.chula.ac.th (V.L.); anusak.k@chula.ac.th (A.K.); 3 Department of Pathology, Faculty of Veterinary Science, Chulalongkorn University, Pratumwan, Bangkok 10330, Thailand; kasem.r@chula.ac.th; 4 Department of Pharmacology and Physiology, Faculty of Pharmaceutical Sciences, Chulalongkorn University, Bangkok 10330, Thailand; 5 Nanovi Ltd., 227-228 Gloucester Road, Wanchai, Hong Kong; contact.drjovian@gmail.com

**Keywords:** dermal fillers, hyaluronic acid, polylactic acid, embolism, rabbits

## Abstract

(1) Background: Hybrid injectable biomaterials incorporating poly-D,L-lactic acid (PDLLA) and hyaluronic acid (HA) have emerged as an important class of biostimulators in aesthetic medicine, combining immediate soft-tissue augmentation with sustained collagen biostimulation. Although inadvertent intravascular injection is uncommon, it may lead to severe vascular complications. However, experimental data on the intravascular behaviour of PDLLA-HA hybrid biostimulators remain limited. This study evaluated vascular flow changes, countercurrent flow, and tissue response following intra-arterial injection of Juvelook^®^ and Juvelook Volume^®^ (VAIM Co., Ltd., Seoul, Republic of Korea) in a rabbit ear model. (2) Methods: A total of 112 rabbits (56 animals per product) were studied using an identical protocol. Each rabbit received bilateral intra-arterial injections of either 0.1 or 0.2 mL of PDLLA-HA hybrid biostimulator prepared at the manufacturer’s recommended 6 mL reconstitution or at serial dilutions ranging from 12 to 42 mL. Immediate vascular flow, countercurrent flow, macroscopic skin changes, and histopathological findings were evaluated over a 7-day observation period. (3) Results: At an injection volume of 0.1 mL, both products dispersed throughout the arterial system without persistent vascular occlusion or tissue necrosis. At 0.2 mL, transient vascular occlusion occurred in the standard (6 mL) and 12 mL dilution groups, with spontaneous restoration of blood flow within 5 min. Countercurrent flow was observed only in the Juvelook Volume^®^ (VAIM Co., Ltd.) cohort under these conditions. No skin necrosis, intravascular thrombosis, or embolisation was observed. Histopathological examination demonstrated preserved vascular integrity, with only focal perivascular foreign-body reactions consistent with injectable biomaterials. (4) Conclusions: Neither biostimulator caused persistent vascular occlusion or clinically significant tissue injury under the experimental conditions investigated. Injection volume and dilution influenced transient vascular flow disturbance and countercurrent behaviour. These findings provide experimental evidence that improves current understanding of the short-term intravascular behaviour of PDLLA-HA hybrid biostimulators and may contribute to future assessments of vascular safety.

## 1. Introduction

Hybrid injectable biomaterials combining poly-D,L-lactic acid with hyaluronic acid (PDLLA-HA) have emerged as a new generation of biostimulators that combine immediate soft tissue augmentation with sustained collagen biostimulation and extracellular matrix remodelling. Owing to their dual mechanism of action, PDLLA-HA hybrid biostimulators are increasingly utilised in aesthetic medicine for facial rejuvenation, acne scar treatment, contour enhancement, and soft tissue restoration. In these hybrid formulations, HA provides immediate volumisation through tissue hydration, whereas biodegradable PDLLA microspheres stimulate fibroblast activation, neocollagenesis, and progressive dermal remodelling, thereby contributing to longer-lasting clinical outcomes. In addition to their clinical applications, PDLLA-based biomaterials have attracted increasing interest because of their favourable biodegradability, biocompatibility, and regenerative potential [1,2,3,4,5].

Beyond collagen stimulation, accumulating experimental evidence has demonstrated that PDLLA-based biomaterials exert broader biological effects, including the modulation of macrophage activity, promotion of angiogenesis, extracellular matrix organisation, adipogenesis, adipose-derived stem cell activation, and hair follicle regeneration in aged skin. These biological responses contribute to tissue regeneration through a controlled foreign body reaction rather than simple volumisation, distinguishing PDLLA-based biostimulators from conventional fillers that function primarily through volume replacement. Recent reviews have further highlighted the role of biodegradable polymeric biomaterials in regulating cytokine signalling and tissue remodelling, supporting their expanding applications in regenerative aesthetics [4,5,6,7,8,9].

Despite favourable clinical outcomes and an excellent overall safety profile, inadvertent intra-arterial injection remains one of the most serious complications associated with injectable biomaterials. Although uncommon, vascular complications may result in tissue ischaemia, skin necrosis, irreversible visual impairment, or cerebrovascular events. The likelihood and severity of vascular injury are influenced by multiple factors, including injection technique, vascular anatomy, injection pressure, bolus volume, rheological properties, particle size distribution, and the physicochemical properties of the injected material. While the vascular safety of HA fillers has been extensively investigated, comparatively little experimental evidence is available regarding the intravascular behaviour of PDLLA-containing hybrid biostimulators. Recent expert consensus has also emphasised the need for further experimental evidence to better characterise the vascular safety profile of PDLLA-HA hybrid biostimulators and inform strategies for preventing and managing vascular complications [1,10,11,12,13,14,15,16].

Juvelook^®^ and Juvelook Volume^®^ (VAIM Co., Ltd.) are commercially available PDLLA-HA hybrid biostimulators that share the same PDLLA-to-HA ratio (85:15) but differ in total PDLLA content, particle size distribution, and intended injection depth. Juvelook^®^ (VAIM Co., Ltd.) contains smaller PDLLA microspheres (approximately 20–40 μm) and is primarily used for superficial dermal indications, whereas Juvelook Volume^®^ (VAIM Co., Ltd.) contains larger microspheres (approximately 40–60 μm), a higher total PDLLA content, and is designed for deeper soft tissue augmentation. These formulation-specific physicochemical differences may influence injectability, particle dispersion, rheological behaviour, and vascular responses following inadvertent intra-arterial injection and therefore warrant experimental investigation [2,3,16].

The present study investigated vascular flow disturbance, countercurrent flow, and tissue responses following controlled intra-arterial injection of Juvelook^®^ and Juvelook Volume^®^ (VAIM Co., Ltd.) using a rabbit ear model. The effects of injection volume and dilution on vascular dynamics and histopathological outcomes were specifically evaluated to generate experimental evidence relevant to vascular safety. By characterising the short-term intravascular behaviour of PDLLA-HA hybrid biostimulators under controlled experimental conditions, this study provides preclinical observations of their vascular responses and contributes to the growing evidence regarding their vascular behaviour.

## 2. Materials and Methods

### 2.1. Study Design

This study comprised two product-specific rabbit ear experiments conducted at the Chulalongkorn University Laboratory Animal Center (CULAC) using an identical predefined experimental protocol. Juvelook^®^ and Juvelook Volume^®^ (VAIM Co., Ltd.) were evaluated in separate cohorts (*n* = 56 rabbits per product), with identical housing conditions, injection procedures, dilution protocols, observation time points, and histopathological assessments.

A total of 112 healthy white rabbits (male and female; 12–16 weeks of age) were included in this study. Animals were housed individually in standard rabbit cages with ad libitum access to food and water under a 12 h light/12 h dark cycle in a temperature-controlled environment (20 ± 1 °C) with a relative humidity of 50 ± 10%.

All experimental procedures were conducted in accordance with institutional guidelines for the care and use of laboratory animals and were approved by the Institutional Animal Care and Use Committee (IACUC) of the Chulalongkorn University Laboratory Animal Center (Protocol Number 237039).

Each rabbit received bilateral intra-arterial injections into the central ear artery (CEA), with 0.1 mL administered to one ear and 0.2 mL administered to the contralateral ear, thereby allowing paired within-animal comparisons. Each experimental group comprised seven rabbits (n = 7), corresponding to 14 ears per treatment group. This paired design was adopted to minimise inter-animal variability and improve the consistency of comparisons between injection conditions. Given the localised nature of the rabbit ear model, crossover effects between the two ears were considered negligible.

### 2.2. Injectable Materials

Juvelook^®^ (VAIM Co., Ltd.) contains PDLLA microspheres with a particle size range of 20–40 µm and contains 42.5 mg of PDLLA and 7.5 mg of non-crosslinked HA per vial. Juvelook Volume^®^ (VAIM Co., Ltd.) contains larger PDLLA microspheres (40–60 µm) with 170 mg of PDLLA and 30 mg of non-crosslinked HA per vial. The standard concentration was defined as the formulation reconstituted according to the manufacturer’s instructions. Briefly, the lyophilised powder was reconstituted with 6 mL of sterile normal saline (0.9% NaCl) to obtain the standard working concentration prior to further dilution.

### 2.3. Experimental Groups and Dilution

For each product cohort, rabbits were randomly allocated into eight experimental groups comprising a control group (0.9% NaCl) and seven PDLLA-HA groups prepared using the standard reconstitution (1:0; 6 mL) or serial dilutions of 1:1 (12 mL), 1:2 (18 mL), 1:3 (24 mL), 1:4 (30 mL), 1:5 (36 mL), and 1:6 (42 mL). The relationship between dilution ratio and final reconstitution volume is summarised in Table 1.

Each experimental group comprised seven rabbits (14 ears). Every rabbit received bilateral intra-arterial injections into the CEA, with 0.1 mL administered to one ear and 0.2 mL administered to the contralateral ear. The assigned solution (0.9% NaCl or PDLLA-HA biostimulator prepared at the designated dilution) was injected according to the allocated experimental group.

**Figure 1:**
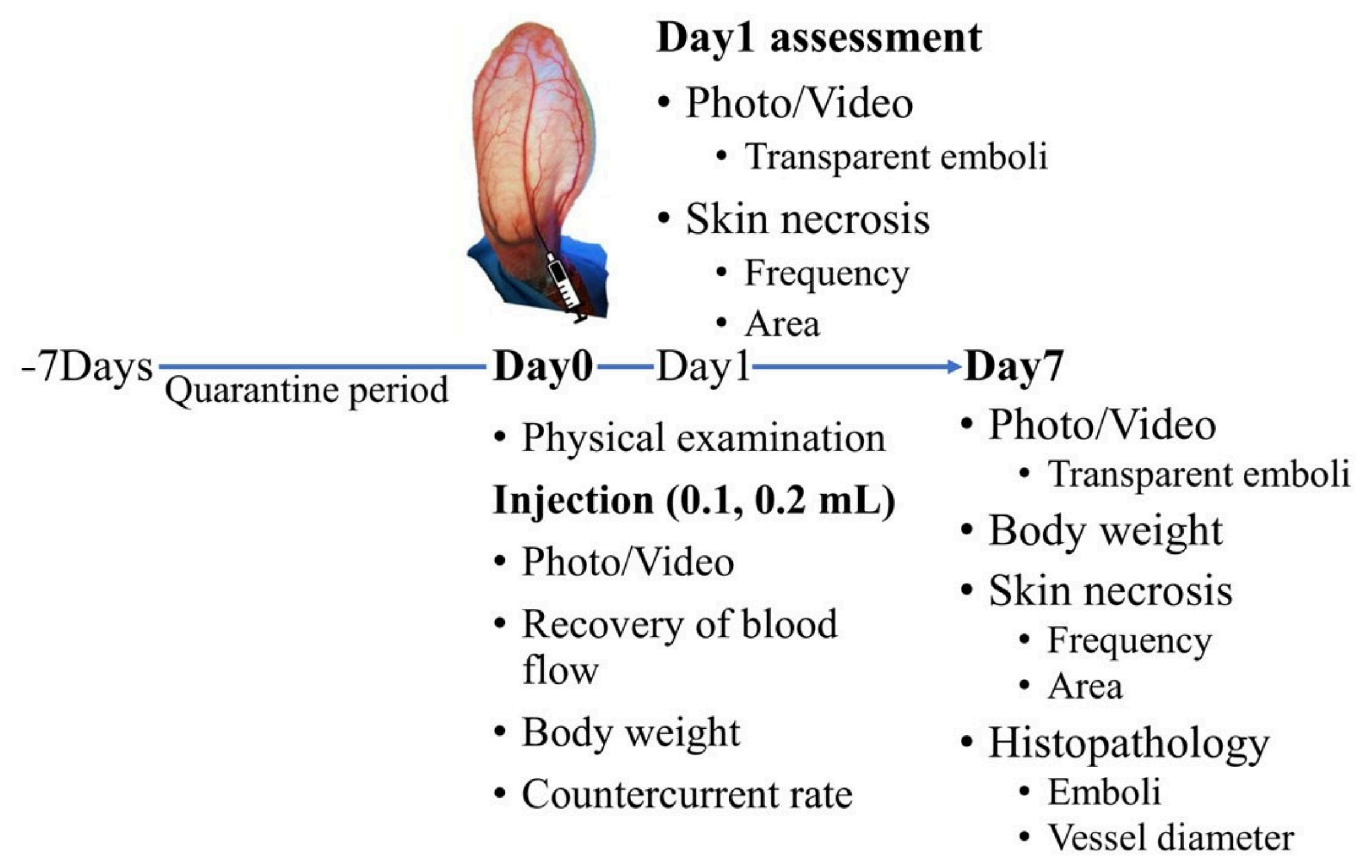
Schematic overview of the experimental study design.

The experimental design for each product cohort is summarised in Figure 1.

Rabbits were allocated to either the Juvelook^®^ or Juvelook Volume^®^ (VAIM Co., Ltd.) cohort and subsequently assigned to one of eight treatment groups comprising a normal saline control or PDLLA-HA biostimulator prepared at standard concentration (6 mL) or serial dilutions of 12, 18, 24, 30, 36, or 42 mL. Each rabbit received bilateral intra-arterial injections of 0.1 mL and 0.2 mL into contralateral ears.

### 2.4. Injection Procedure

On the day of the experiment, rabbits were gently restrained using a rabbit restrainer. The injection site on each ear was shaved, and topical lidocaine spray was applied to provide local anaesthesia. After 15–20 min, the skin was cleaned with sterile saline-soaked gauze. The ventral surface of the rabbit ear was illuminated using a high-intensity light source to facilitate visualisation of the vasculature.

**Figure 2:**
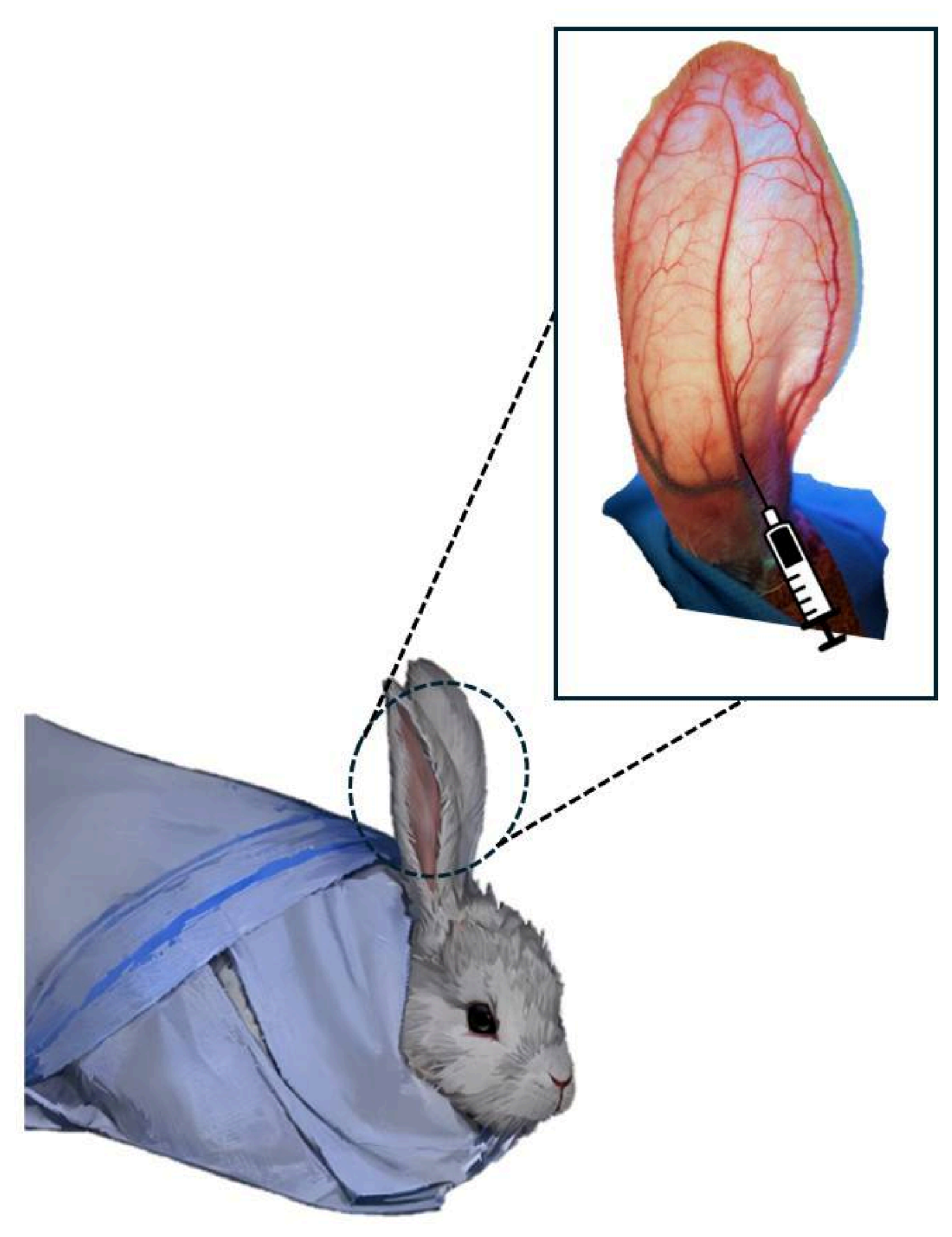
Schematic diagram of rabbit ear vasculature and injection site. The central ear artery (CEA) and central ear vein (CEV) are illustrated. Intra-arterial injections were administered approximately 1 cm proximal to the medial branch of the CEV, corresponding to 4–5 cm from the base of the rabbit ear.

The assigned solution was injected into the main trunk of the CEA using a 26-gauge needle. The injection site was located approximately 1 cm proximal to the medial branch of the central ear vein (CEV), corresponding to approximately 4–5 cm from the base of the rabbit ear (Figure 2). All injections were performed at a controlled rate of approximately 0.2 mL/min to minimise variability between animals.

### 2.5. Observations

Vascular blood flow was observed under direct illumination and digitally recorded immediately before injection and continuously for the first 5 min following injection.

Immediate vascular responses were categorised into three predefined groups: Category I, uninterrupted arterial flow with normal dispersion of the injected material and no evidence of vascular obstruction; Category II, transient arterial occlusion with complete restoration of blood flow within 5 min; and Category III, persistent arterial occlusion with incomplete restoration of blood flow in the main branch of the CEA beyond 5 min.

Countercurrent flow was defined as retrograde reflux of the injected material beyond the injection site and was expressed as the percentage of ears demonstrating this phenomenon.

Animals were examined on post-injection days 1 and 7 for changes in blood flow, skin colour, evidence of transparent emboli, skin necrosis, and the extent of any necrotic lesions. Representative clinical photographs were obtained at each observation time point.

### 2.6. Histopathology

**Figure 3:**
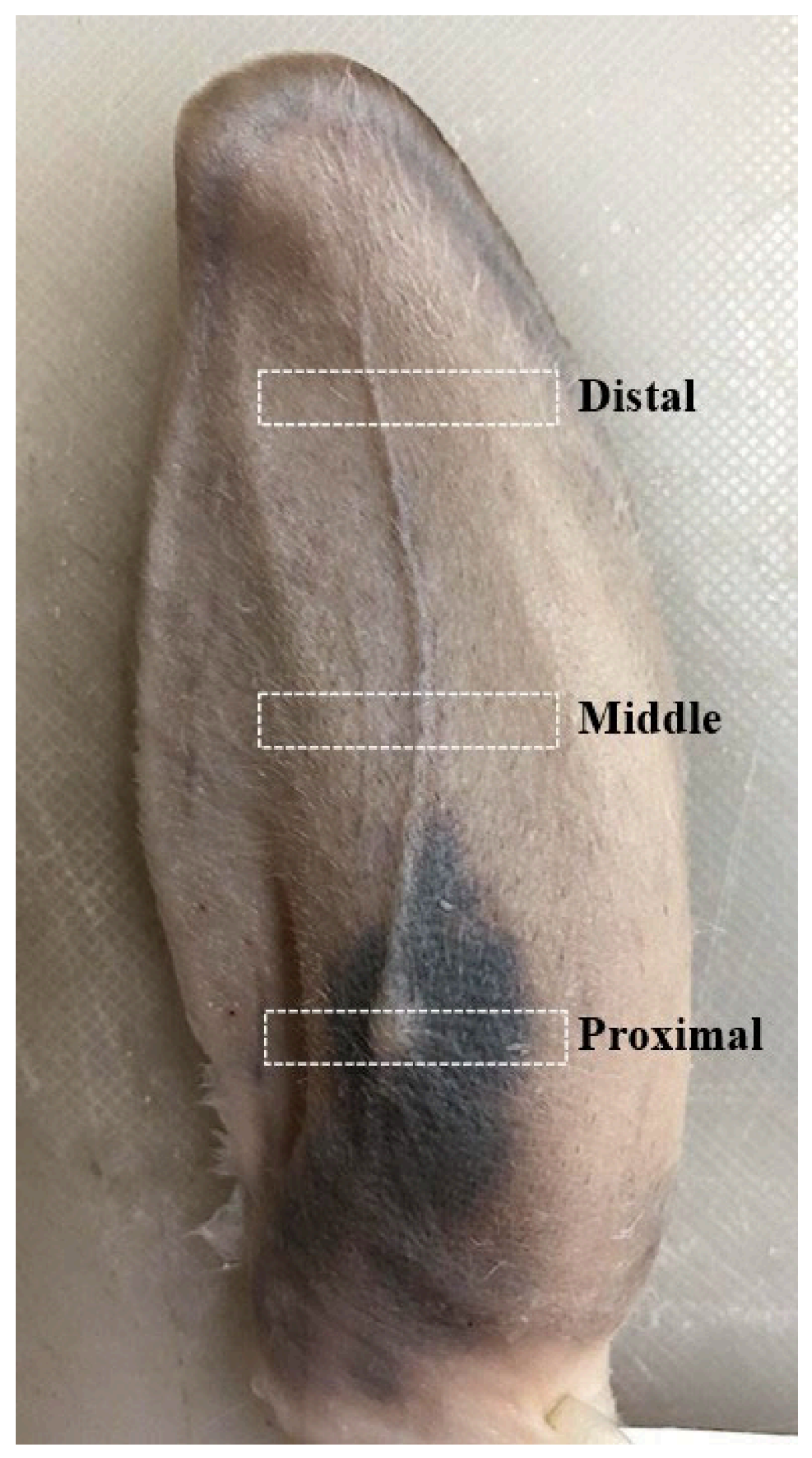
Schematic illustration of tissue sampling for histopathological analysis. The rabbit ear was divided into proximal, middle, and distal segments along the course of the central ear artery (CEA) for histopathological evaluation. Tissue specimens from each segment were processed independently following fixation.

On day 7 after injection, the entire ear pinna was harvested and fixed in 10% neutral-buffered formalin. Following fixation, each specimen was divided into three anatomical segments along the course of the central artery, designated as the proximal, middle, and distal segments (Figure 3). Tissue samples were routinely processed, embedded in paraffin, sectioned at a thickness of 4 µm, and stained with haematoxylin and eosin (H&E). Outcome assessors were not blinded to treatment allocation during the macroscopic or histopathological evaluations.

**Figure 4:**
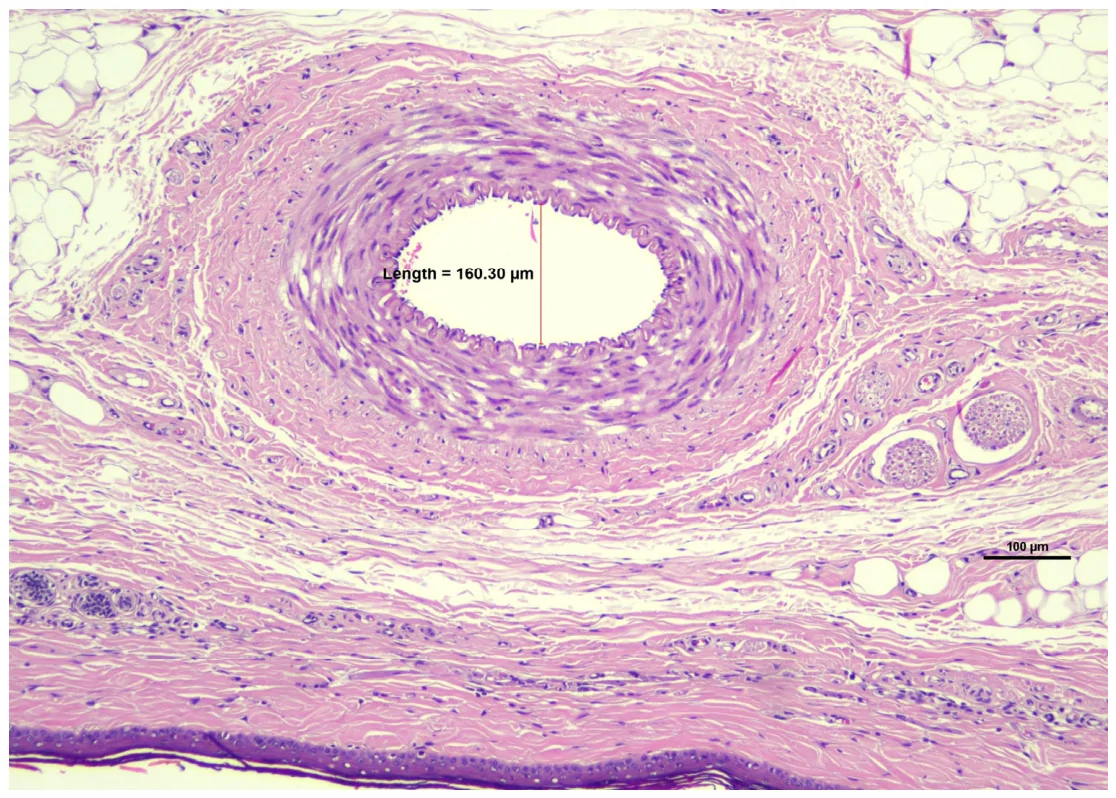
Measurement of the largest blood vessel in the distal ear segment. Representative histological section demonstrating digital measurement of the largest blood vessel diameter within the distal ear segment using NIS-Elements BR software (version 5.21.00; Nikon Corporation, Tokyo, Japan).

Histopathological examination was performed using a Nikon Eclipse Ci light microscope (Nikon Corporation, Tokyo, Japan), and representative images were captured using a Nikon DS-Ri2 camera (Figure 4). The diameter of the largest blood vessel within the distal segment was measured using digital image analysis with NIS-Elements BR software (version 5.21.00; Nikon Corporation, Tokyo, Japan).

### 2.7. Statistical Analysis

Continuous variables are presented as mean ± standard deviation (SD). Categorical variables are presented as frequencies and percentages. Fisher’s exact test was used for comparisons of categorical outcomes where expected cell counts were small. A non-parametric test was used to compare the area of skin lesions and the diameter of the largest blood vessel between groups. Changes in body weight were analysed using two-way analysis of variance (ANOVA), with treatment/dilution group and injection volume as factors. The main effects of treatment/dilution group and injection volume, as well as their interaction, were assessed. A two-sided *p*-value < 0.05 was considered statistically significant.

Because both ears of each rabbit were included in the study, observations obtained from contralateral ears were not statistically independent. Consequently, the 0.1 mL and 0.2 mL injection volumes were considered paired within-animal observations. Comparisons between separate treatment groups and their corresponding saline controls were performed using Fisher’s exact test where appropriate.

## 3. Results

### 3.1. Body Weight Changes

**Figure 5:**
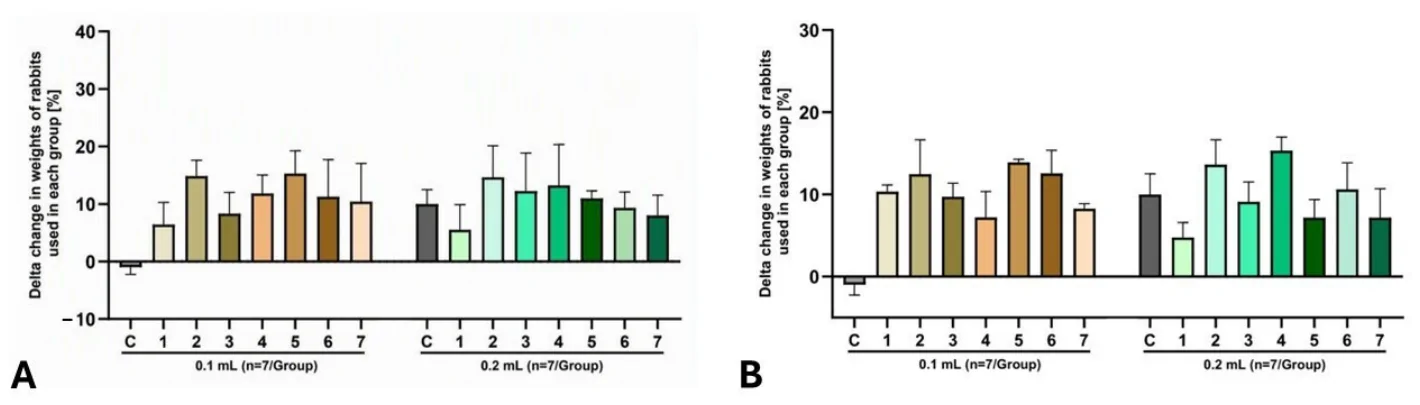
(**A**) Percentage change in body weight following intra-arterial injection of Juvelook^®^ (*n* = 7/group). (**B**) Percentage change in body weight following intra-arterial injection of Juvelook Volume^®^ (*n* = 7/group). Error bars represent standard deviation (SD).

Body weight was monitored throughout the study to evaluate potential systemic effects following intra-arterial injection. No statistically significant differences in body weight were observed between baseline and day 7 in any experimental group. Rabbits receiving Juvelook^®^ or Juvelook Volume^®^ (VAIM Co., Ltd.) demonstrated only minor fluctuations in body weight, which were comparable to those observed in the corresponding 0.9% NaCl control groups. Similarly, no statistically significant differences were identified between the 0.1 mL and 0.2 mL injection volumes for either product. These findings indicate that neither PDLLA-HA hybrid biostimulator produced detectable systemic effects during the 7-day observation period (Figure 5A,B).

### 3.2. Immediate Vascular Flow Changes

**Figure 6:**
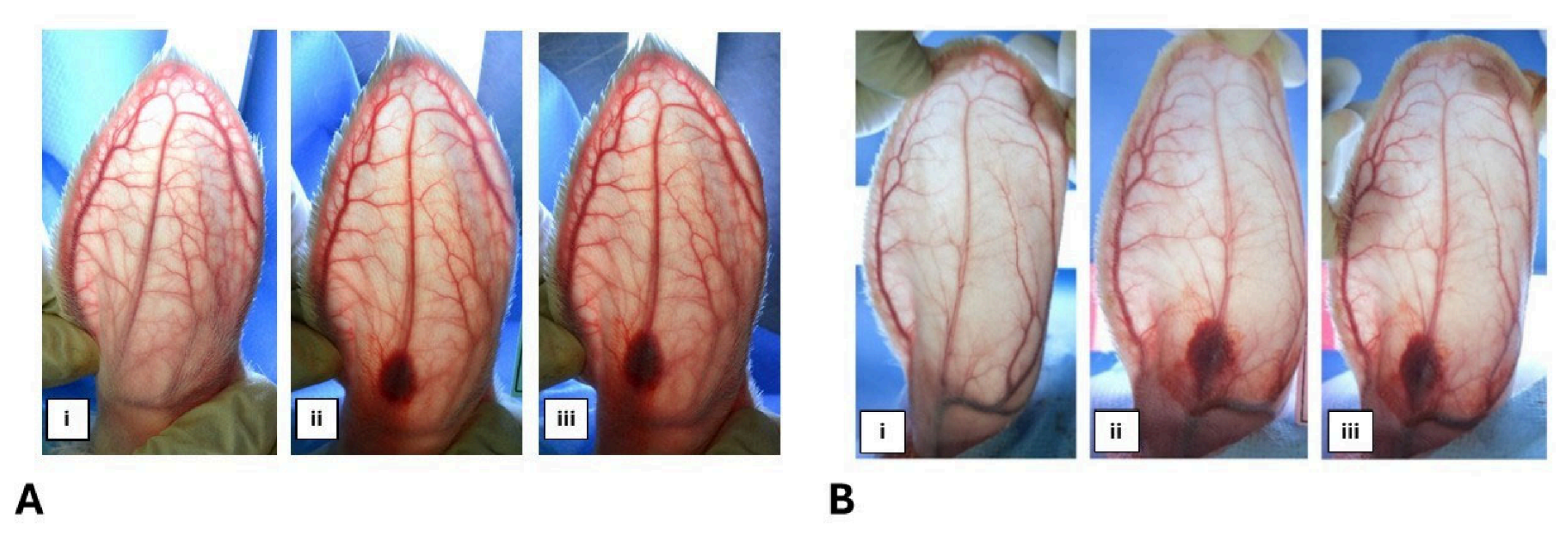
Representative vascular changes in rabbit ears following intra-arterial injection of PDLLA-HA hybrid biostimulators. (**A**) Juvelook® and (**B**) Juvelook Volume® following injection of 0.2 mL. For both products: (i) before injection, showing normal arterial perfusion; (ii) immediately after injection, showing transient vascular occlusion; and (iii) 5 min after injection, showing restoration of arterial blood flow.

Before injection, the vascular anatomy of the rabbit ear was clearly visualised under direct illumination, with the CEA, CEV, and terminal arterial branches readily identifiable (Figure 6). During injection, the PDLLA-HA hybrid biostimulators progressively dispersed throughout the arterial tree via both the main trunk and peripheral branches of the CEA.

In the 0.9% NaCl control groups, all ears demonstrated normal arterial perfusion without evidence of vascular obstruction, irrespective of injection volume. Consequently, 100% of control ears were classified as Category I.

At the 0.1 mL injection volume, both Juvelook^®^ and Juvelook Volume^®^ (VAIM Co., Ltd.) demonstrated smooth intravascular dispersion across all dilution groups without evidence of persistent vascular obstruction.

**Figure 7:**
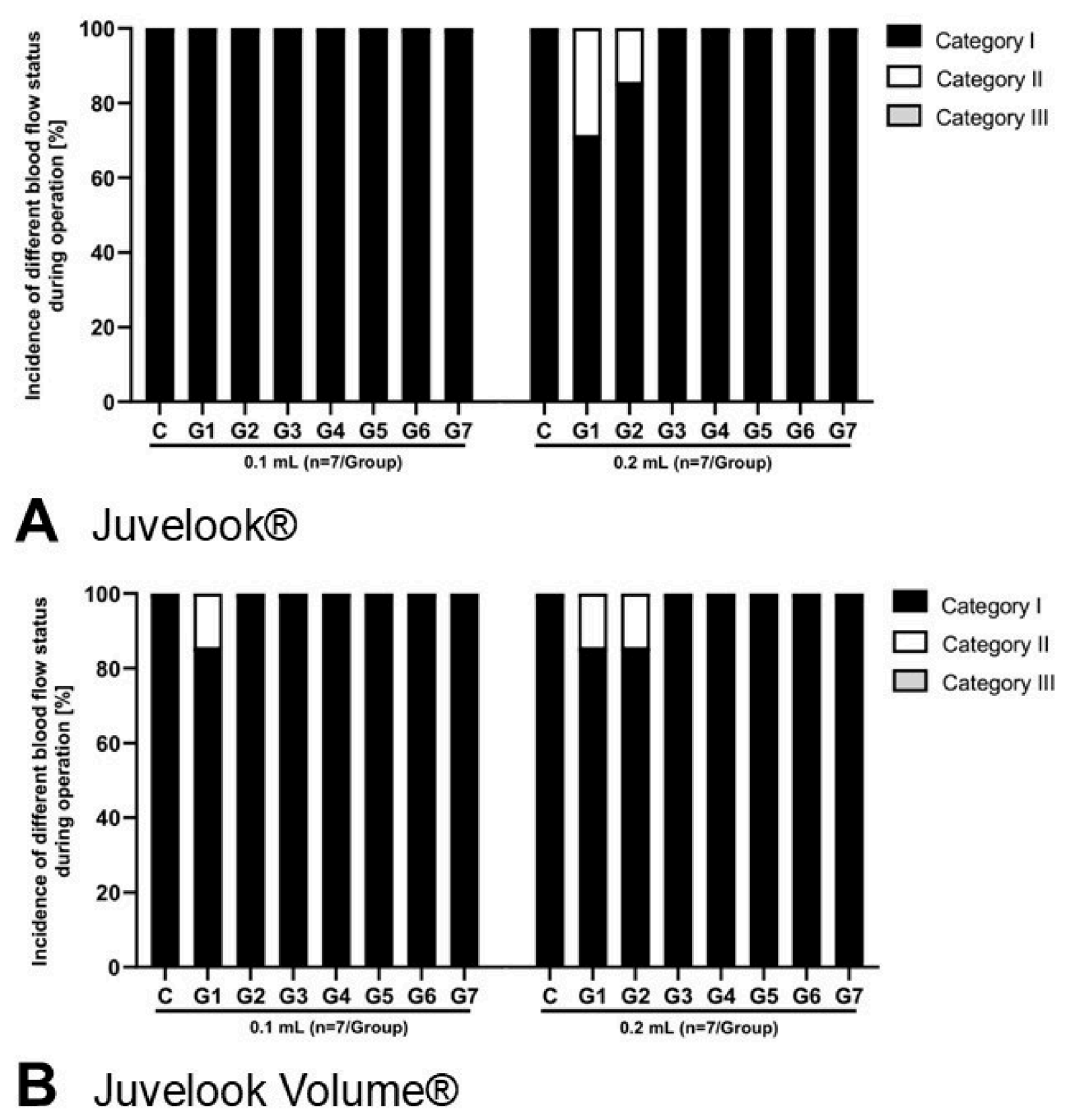
(**A**) Vascular flow status within 5 min following intra-arterial injection of Juvelook^®^ (*n* = 7/group). Category I, normal arterial perfusion; Category II, transient occlusion with restoration of flow within 5 min; Category III, persistent occlusion beyond 5 min. (**B**) Vascular flow status within 5 min following intra-arterial injection of Juvelook Volume^®^ (*n* = 7/group). Category definitions are as described in Figure 7A.

At the 0.2 mL injection volume, transient vascular occlusion was observed in the standard concentration (6 mL) and 12 mL dilution groups for both products. In all affected ears, arterial blood flow was completely restored within 5 min after injection. No persistent vascular occlusion (Category III) was observed in any experimental group. No transient vascular obstruction was observed following further dilution beyond 12 mL (18–42 mL), with uniform intravascular dispersion observed without interruption of blood flow (Figure 6A,B and Figure 7A,B).

### 3.3. Countercurrent Flow

**Figure 8:**
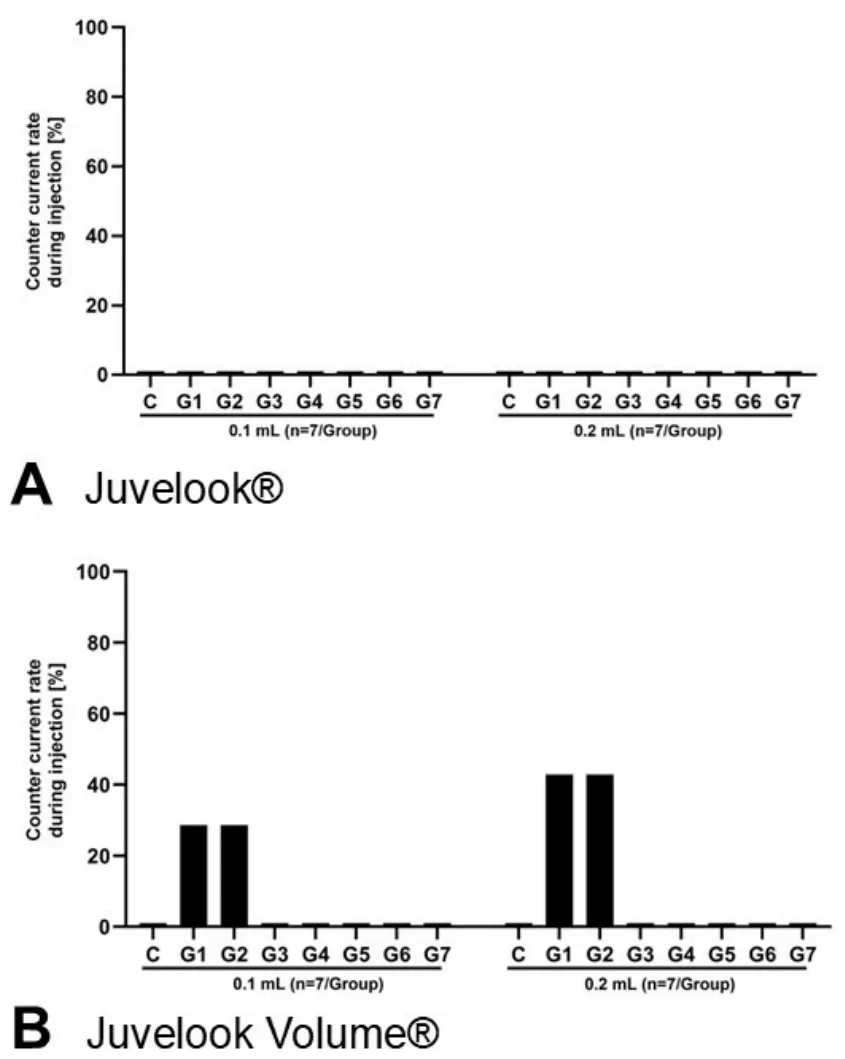
(**A**) Countercurrent flow following intra-arterial injection of Juvelook^®^ at different injection volumes and dilution ratios (*n* = 7/group). No countercurrent flow was observed. (**B**) Countercurrent flow following intra-arterial injection of Juvelook Volume^®^ at different injection volumes and dilution ratios (*n* = 7/group). Countercurrent flow was observed only in the standard concentration (6 mL) and 12 mL dilution groups; differences compared with the corresponding saline controls were not statistically significant by Fisher’s exact test.

Countercurrent flow rates for the control, standard concentration (6 mL), and serial dilution groups at both the 0.1 mL and 0.2 mL injection volumes are presented in Figure 8.

In the Juvelook Volume^®^ (VAIM Co., Ltd.) cohort (Figure 8B), countercurrent flow was observed following injection of the standard concentration at both the 0.1 mL (28.57%) and 0.2 mL (42.86%) injection volumes. Similar countercurrent flow rates were observed in the 12 mL dilution group. Fisher’s exact testing demonstrated no statistically significant difference compared with the corresponding 0.9% NaCl control group at either injection volume (0.1 mL, *p* = 0.462; 0.2 mL, *p* = 0.192). No countercurrent flow was detected following dilution beyond 12 mL (18–42 mL), irrespective of injection volume.

In contrast, no countercurrent flow was observed in any Juvelook^®^ (VAIM Co., Ltd.) group (Figure 8A), regardless of dilution or injection volume. Consequently, no statistically significant differences were identified between Juvelook^®^-treated ears (VAIM Co., Ltd.) and the corresponding 0.9% NaCl control groups.

### 3.4. Skin Necrosis and Embolic Events

**Figure 9:**
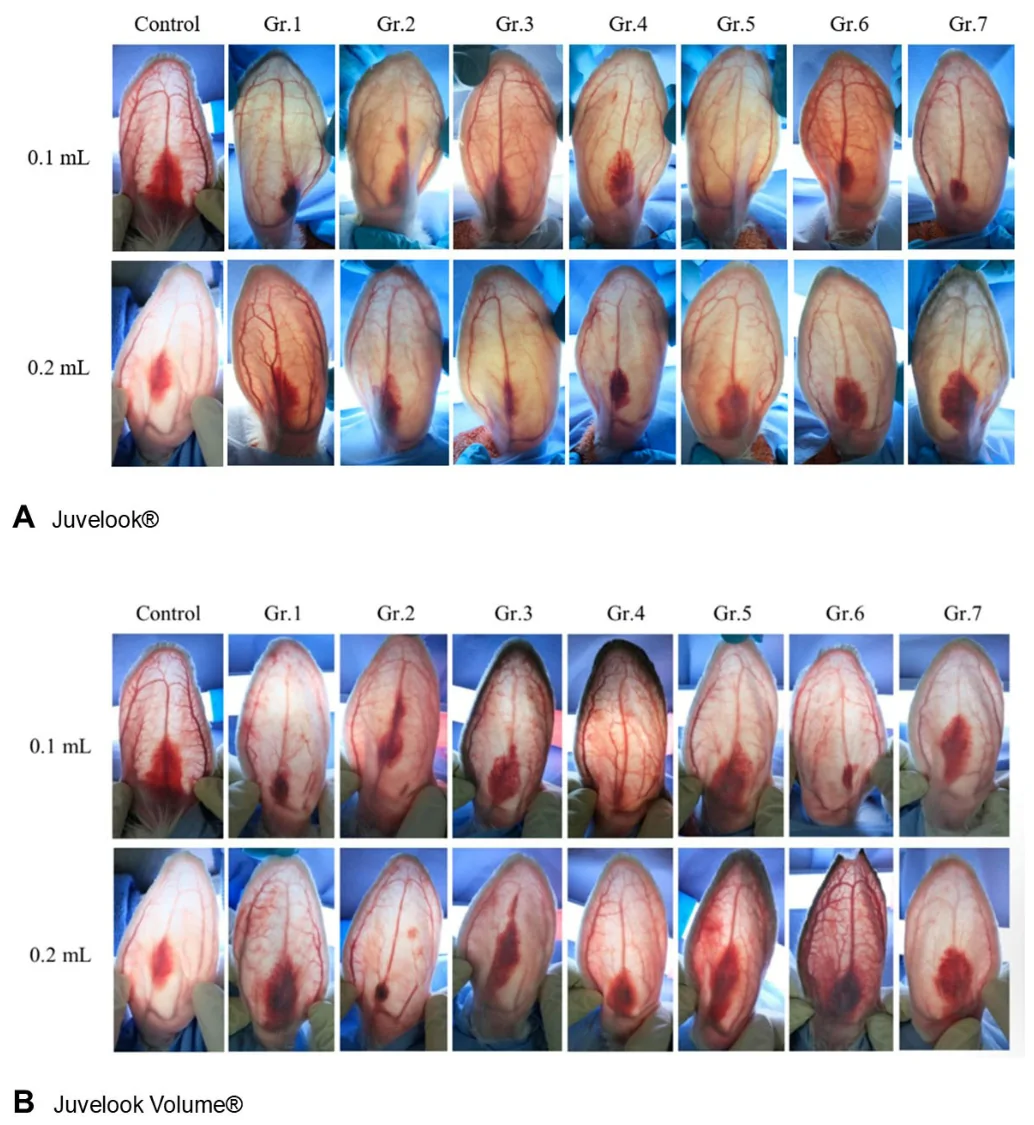
Representative macroscopic appearance of rabbit ears on day 1 following intra-arterial injection. No transparent emboli, skin discoloration, or tissue necrosis were observed in any experimental group.

Macroscopic evaluation of rabbit ears demonstrated no evidence of transparent emboli, skin discoloration, or tissue necrosis in any experimental group at day 1 or day 7 following injection (Figure 9).

**Figure 10:**
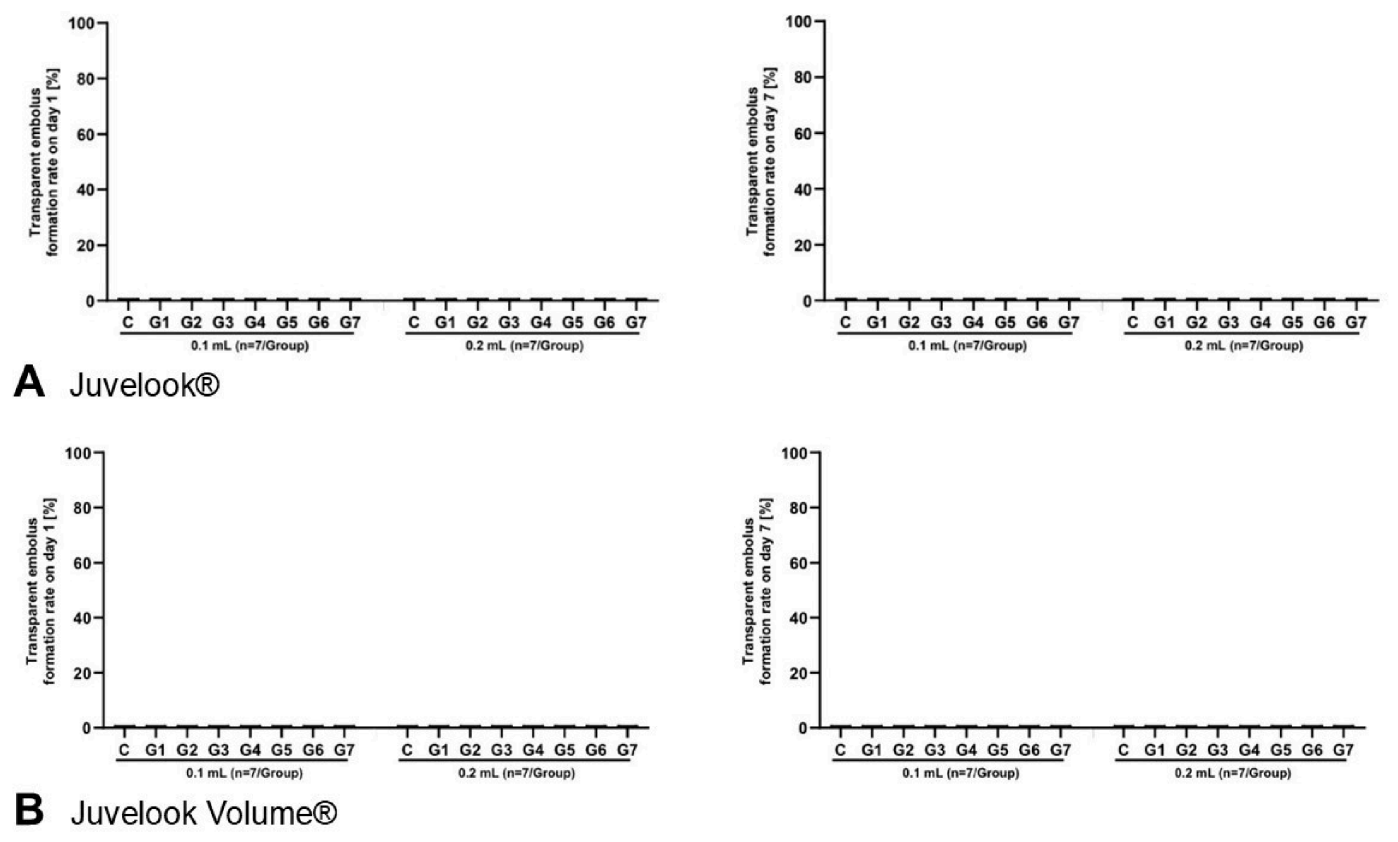
Transparent embolus rate on day 1 and day 7 following intra-arterial injection of Juvelook^®^ and Juvelook Volume^®^ (*n* = 7/group). No transparent emboli were detected at either assessment time point.

**Figure 11:**
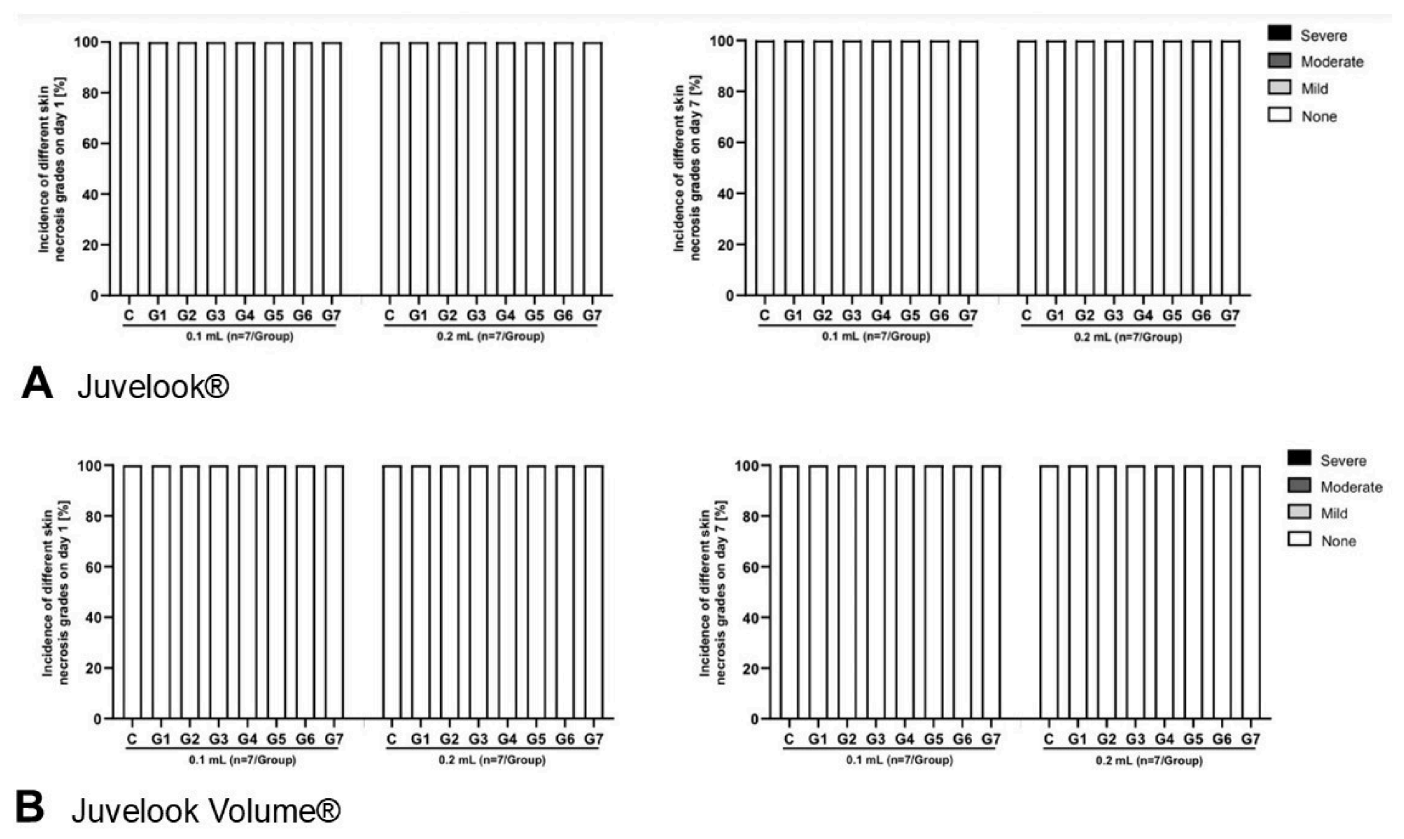
Skin necrosis grade on days 1 and 7 following intra-arterial injection of Juvelook^®^ and Juvelook Volume^®^ (*n* = 7/group). No measurable skin necrosis was observed in any experimental group.

**Figure 12:**
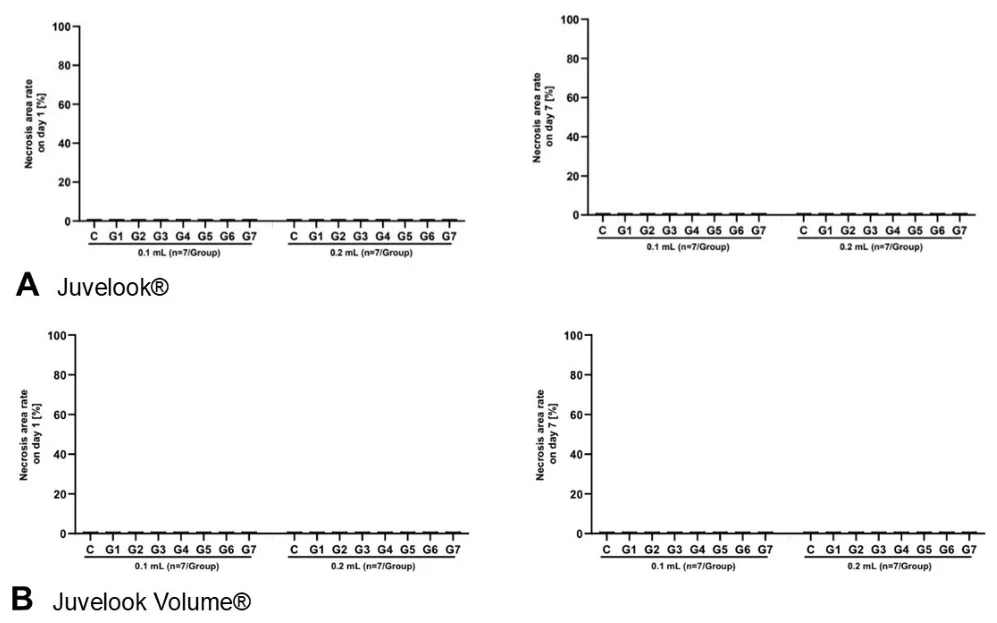
Necrotic area on days 1 and 7 following intra-arterial injection of Juvelook^®^ and Juvelook Volume^®^ (*n* = 7/group). No necrotic lesions were detected at either assessment time point.

Quantitative assessment confirmed the absence of transparent emboli, measurable skin necrosis, and necrotic area in both the Juvelook^®^ and Juvelook Volume^®^ (VAIM Co., Ltd.) cohorts across all injection volumes and dilution conditions (Figure 10, Figure 11 and Figure 12). No clinically observable ischaemic tissue injury was identified during the study period.

### 3.5. Histopathology

Histopathological findings were evaluated separately in the proximal, middle, and distal segments of the rabbit ear 7 days after injection.

**Figure 13:**
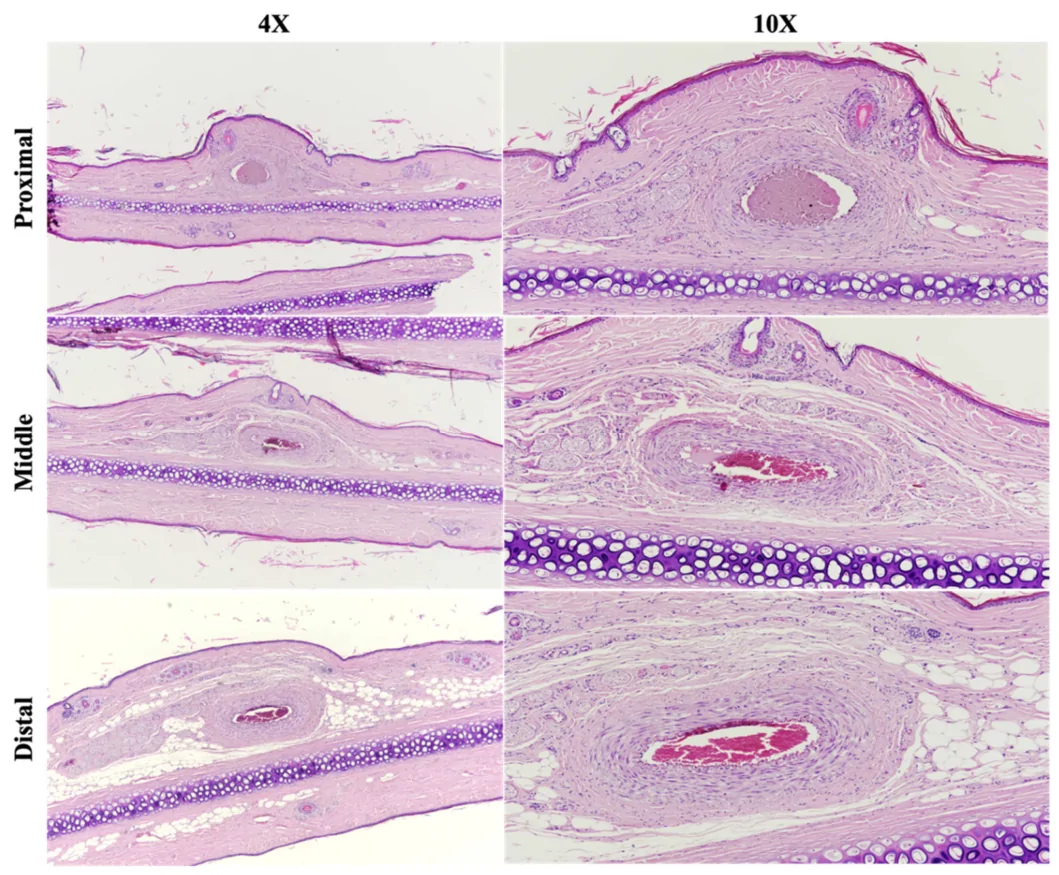
Representative histological findings of rabbit ear tissue following intra-arterial injection of 0.1 mL of 0.9% NaCl. Preserved vascular architecture was observed in the proximal, middle, and distal segments without thrombosis, embolisation, or inflammatory infiltration (H&E staining).

**Figure 14:**
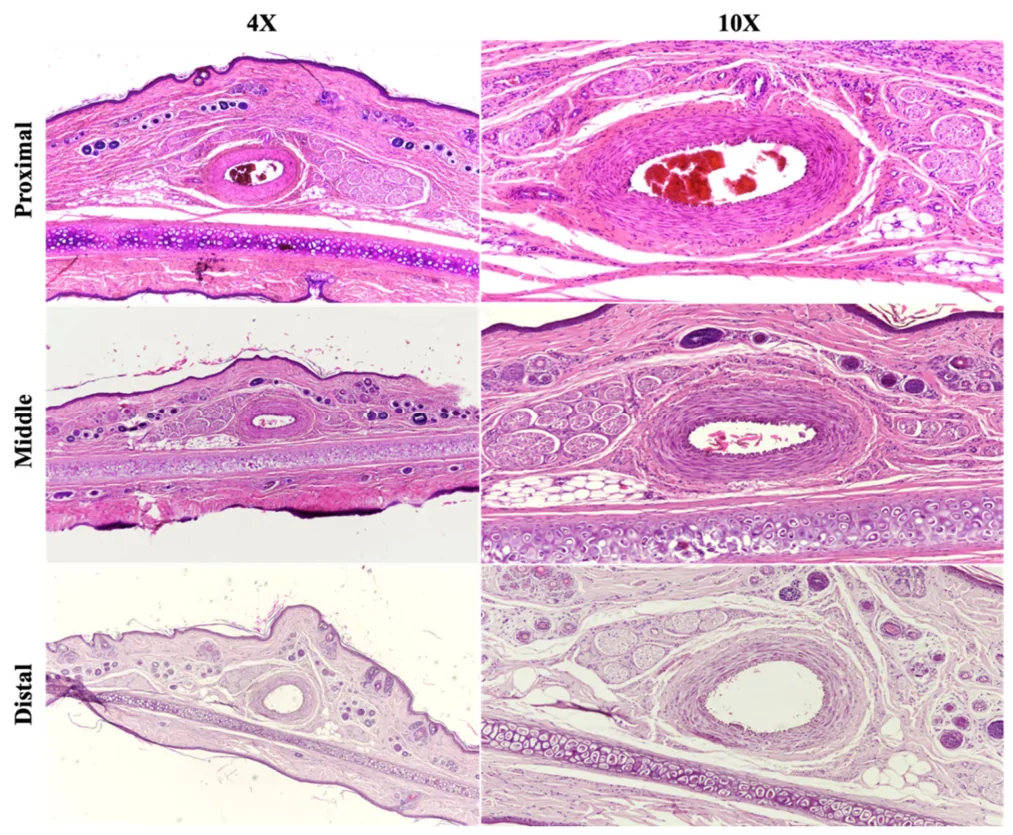
Representative histological findings of rabbit ear tissue following intra-arterial injection of 0.2 mL of 0.9% NaCl. No histopathological abnormalities were identified in any anatomical segment (H&E staining).

In 0.9% NaCl control groups, vascular architecture remained intact throughout all anatomical segments. Representative histological sections obtained following 0.1 mL injection demonstrated preserved vascular morphology without evidence of thrombosis, embolisation, or inflammatory cell infiltration (Figure 13). Similarly, no histopathological abnormalities were identified following 0.2 mL injection (Figure 14). Quantitative measurements of the largest blood vessel within the distal segment are summarised in Table 2.

**Figure 15:**
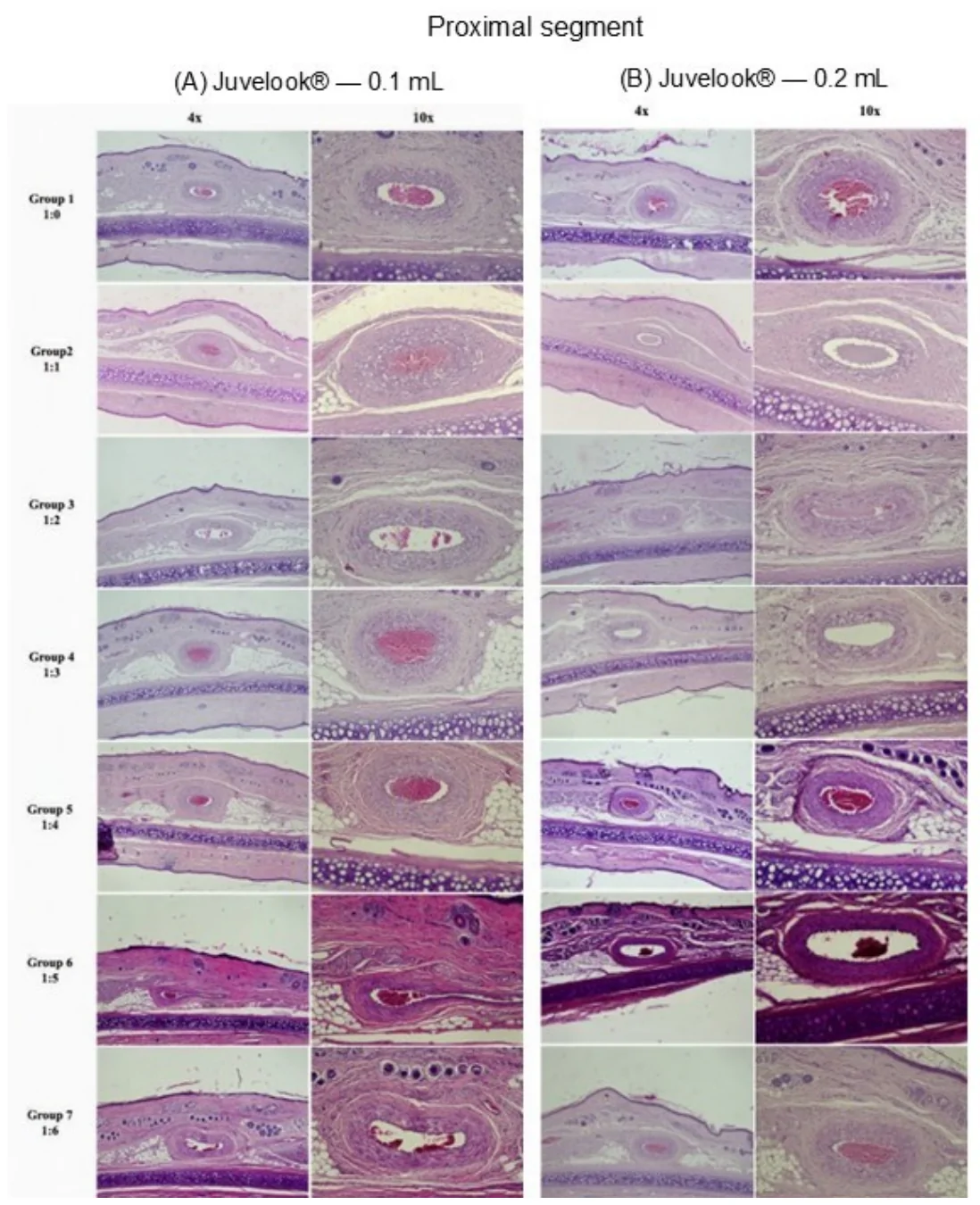
Histological findings of the proximal rabbit ear segments following intra-arterial injection of Juvelook® at different dilution ratios. (**A**) 0.1 mL injection and (**B**) 0.2 mL injection. Groups 1–7 represent the standard preparation (1:0; 6 mL) and serial dilutions of 1:1 (12 mL), 1:2 (18 mL), 1:3 (24 mL), 1:4 (30 mL), 1:5 (36 mL), and 1:6 (42 mL), respectively. Representative sections are shown at 4× and 10× magnification (H&E staining).

**Figure 16:**
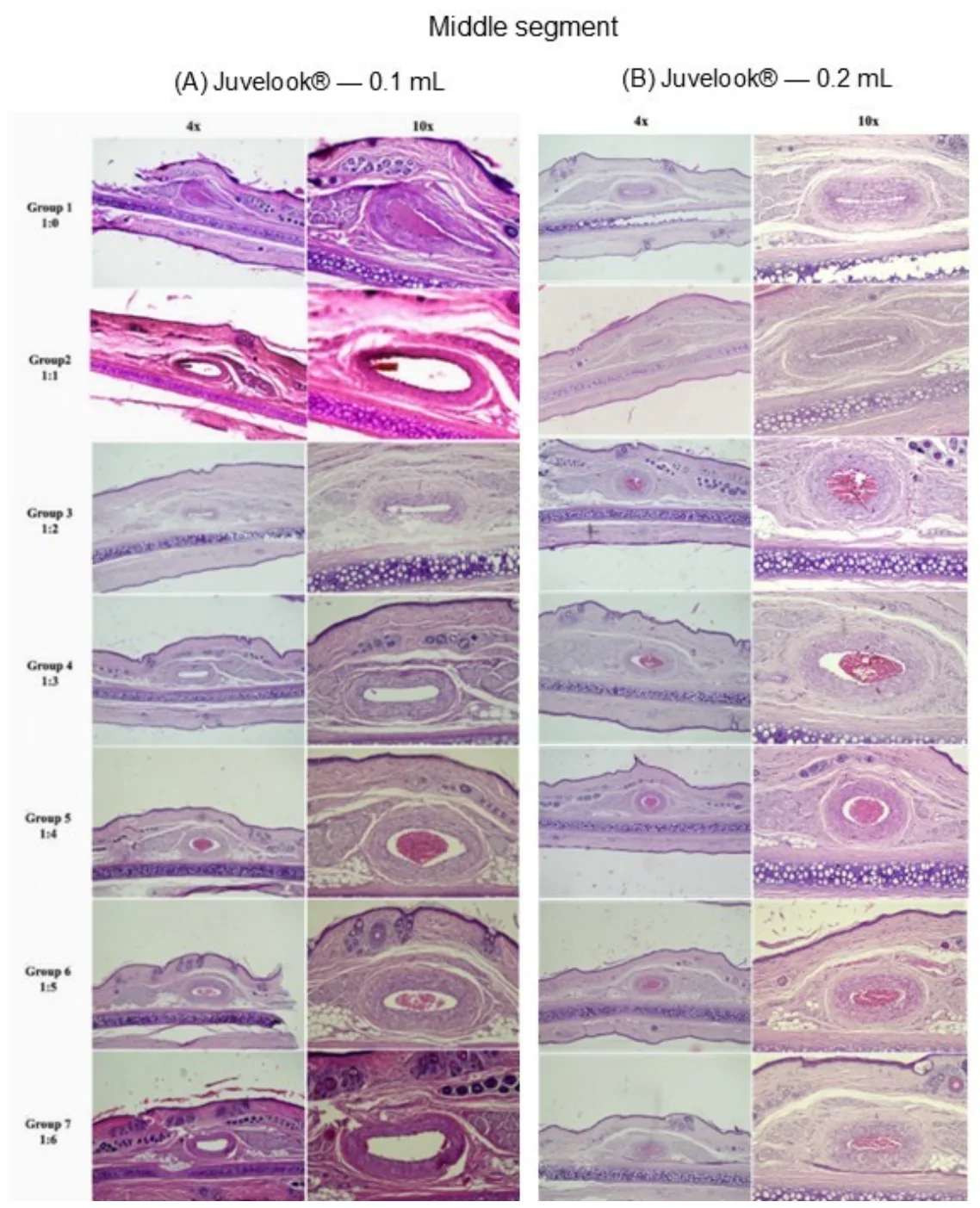
Histological findings of the middle rabbit ear segments following intra-arterial injection of Juvelook® at different dilution ratios. (**A**) 0.1 mL injection and (**B**) 0.2 mL injection. Groups 1–7 represent the standard preparation (1:0; 6 mL) and serial dilutions of 1:1 (12 mL), 1:2 (18 mL), 1:3 (24 mL), 1:4 (30 mL), 1:5 (36 mL), and 1:6 (42 mL), respectively. Representative sections are shown at 4× and 10× magnification (H&E staining).

**Figure 17:**
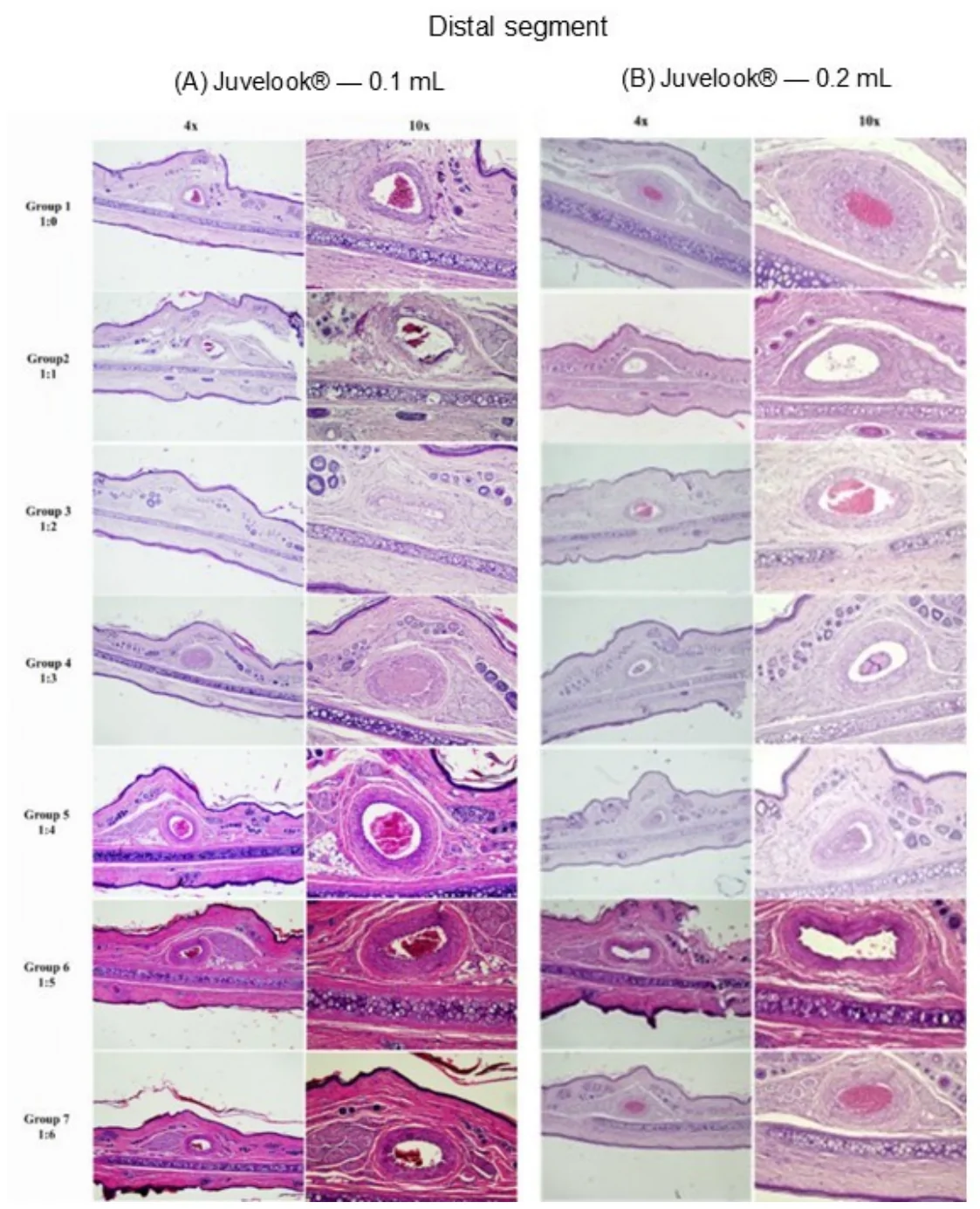
Histological findings of the distal rabbit ear segments following intra-arterial injection of Juvelook® at different dilution ratios. (**A**) 0.1 mL injection and (**B**) 0.2 mL injection. Groups 1–7 represent the standard preparation (1:0; 6 mL) and serial dilutions of 1:1 (12 mL), 1:2 (18 mL), 1:3 (24 mL), 1:4 (30 mL), 1:5 (36 mL), and 1:6 (42 mL), respectively. Representative sections are shown at 4× and 10× magnification (H&E staining).

**Figure 18:**
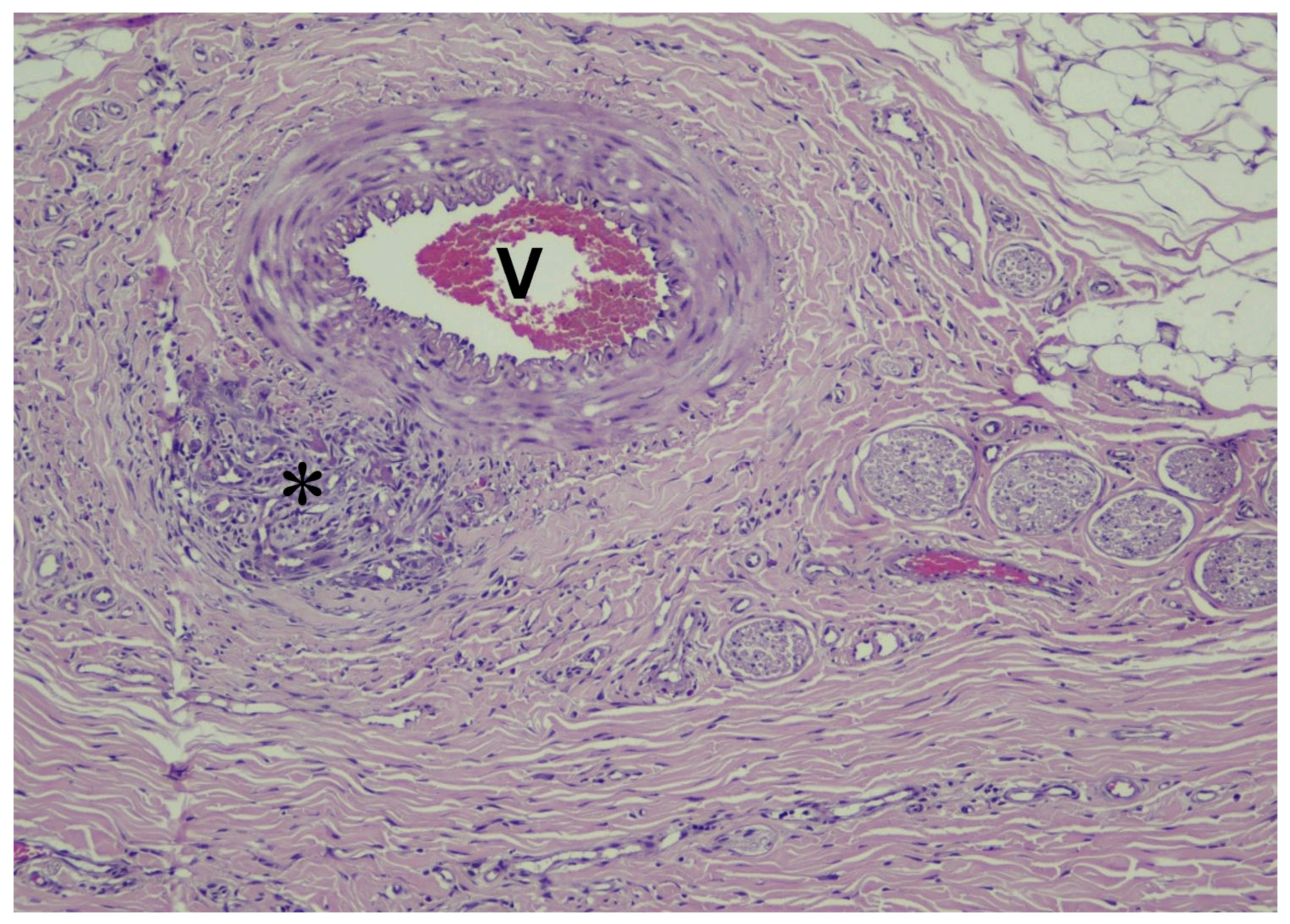
Representative foreign body reaction (*) adjacent to a large vessel (V) in the proximal rabbit ear segment following intra-arterial injection of 0.1 mL Juvelook® at a 1:3 dilution ratio (H&E staining).

Juvelook®-treated (VAIM Co., Ltd.) ears demonstrated generally preserved vascular architecture. Findings from the proximal segments following 0.1 mL and 0.2 mL injections of the standard preparation and serial dilution groups are shown in Figure 15, with corresponding findings from the middle and distal segments shown in Figure 16 and Figure 17, respectively. Mild foreign body reactions characterised by macrophage infiltration and multinucleated giant cells were occasionally observed adjacent to large vessels, predominantly within the proximal segment. These reactions were focal and confined to the perivascular tissue without involvement of the vascular lumen (Figure 18). No intravascular thrombosis or embolic material was identified. Quantitative measurements of the largest blood vessel in the distal segment are summarised in Table 3.

**Figure 19:**
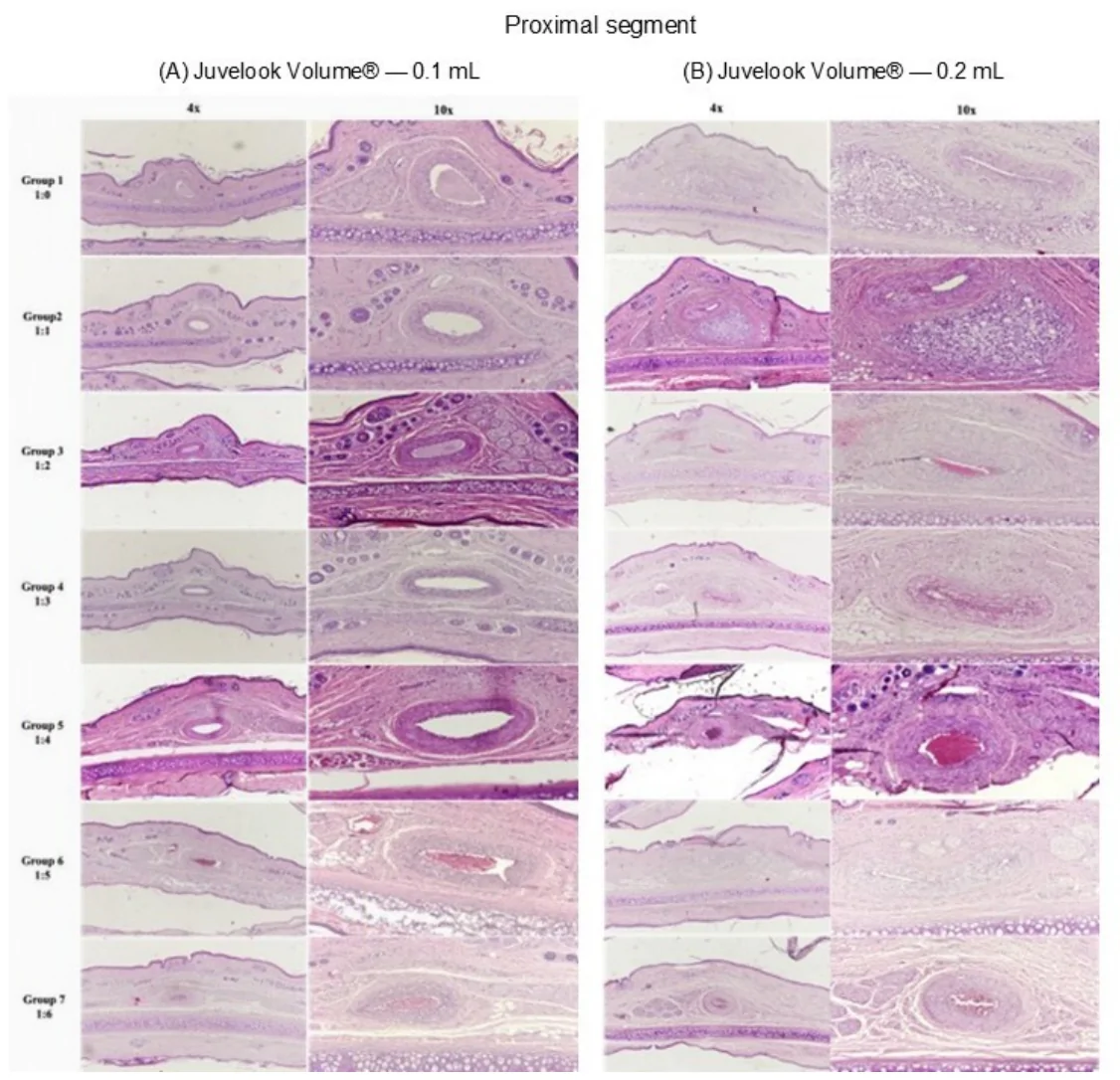
Histological findings of the proximal rabbit ear segments following intra-arterial injection of Juvelook Volume® at different dilution ratios. (**A**) 0.1 mL injection and (**B**) 0.2 mL injection. Groups 1–7 represent the standard preparation (1:0; 6 mL) and serial dilutions of 1:1 (12 mL), 1:2 (18 mL), 1:3 (24 mL), 1:4 (30 mL), 1:5 (36 mL), and 1:6 (42 mL), respectively. Representative sections are shown at 4× and 10× magnification (H&E staining).

**Figure 20:**
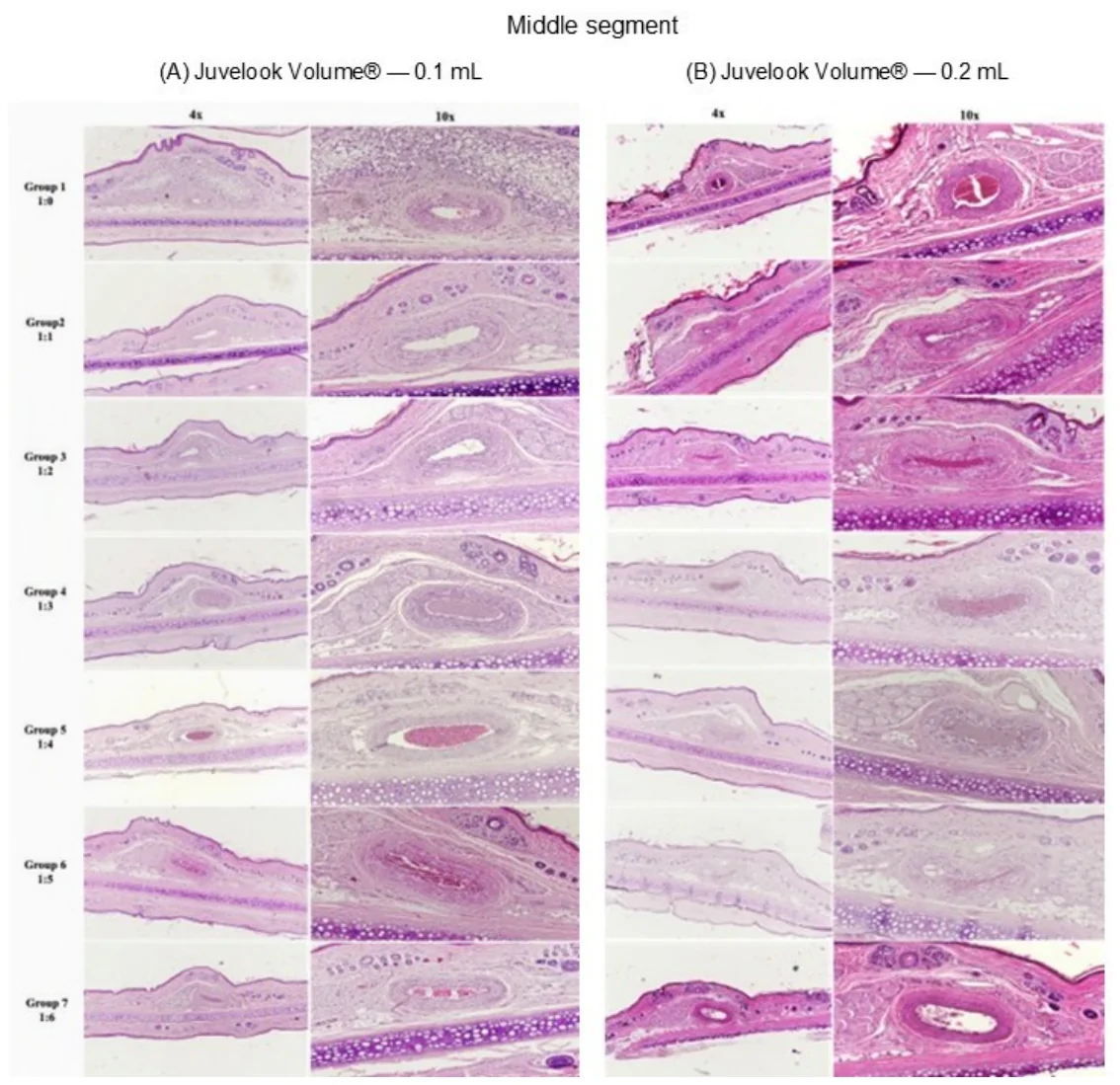
Histological findings of the middle rabbit ear segments following intra-arterial injection of Juvelook Volume® at different dilution ratios. (**A**) 0.1 mL injection and (**B**) 0.2 mL injection. Groups 1–7 represent the standard preparation (1:0; 6 mL) and serial dilutions of 1:1 (12 mL), 1:2 (18 mL), 1:3 (24 mL), 1:4 (30 mL), 1:5 (36 mL), and 1:6 (42 mL), respectively. Representative sections are shown at 4× and 10× magnification (H&E staining).

**Figure 21:**
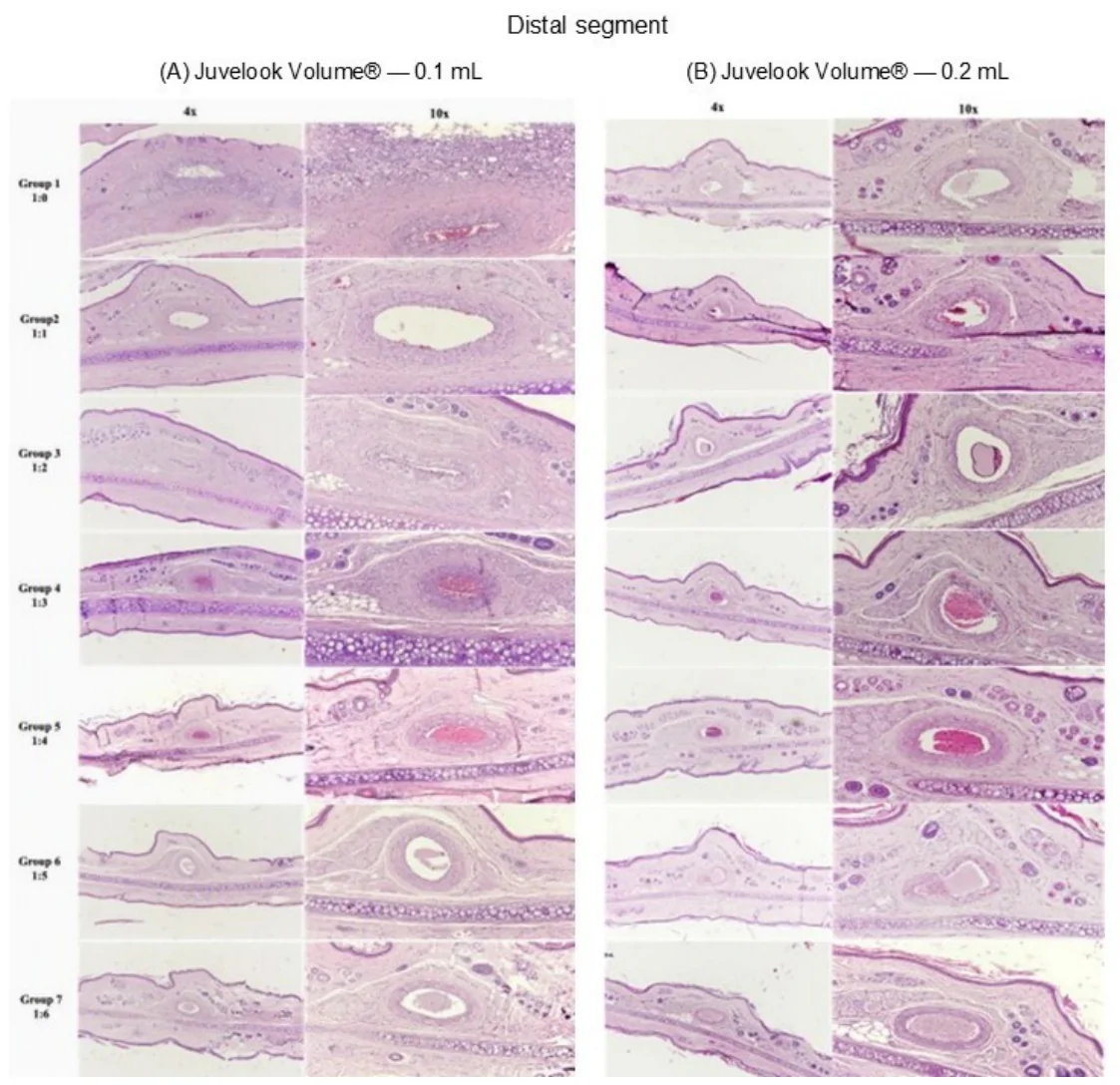
Histological findings of the distal rabbit ear segments following intra-arterial injection of Juvelook Volume® at different dilution ratios. (**A**) 0.1 mL injection and (**B**) 0.2 mL injection. Groups 1–7 represent the standard preparation (1:0; 6 mL) and serial dilutions of 1:1 (12 mL), 1:2 (18 mL), 1:3 (24 mL), 1:4 (30 mL), 1:5 (36 mL), and 1:6 (42 mL), respectively. Representative sections are shown at 4× and 10× magnification (H&E staining).

**Figure 22:**
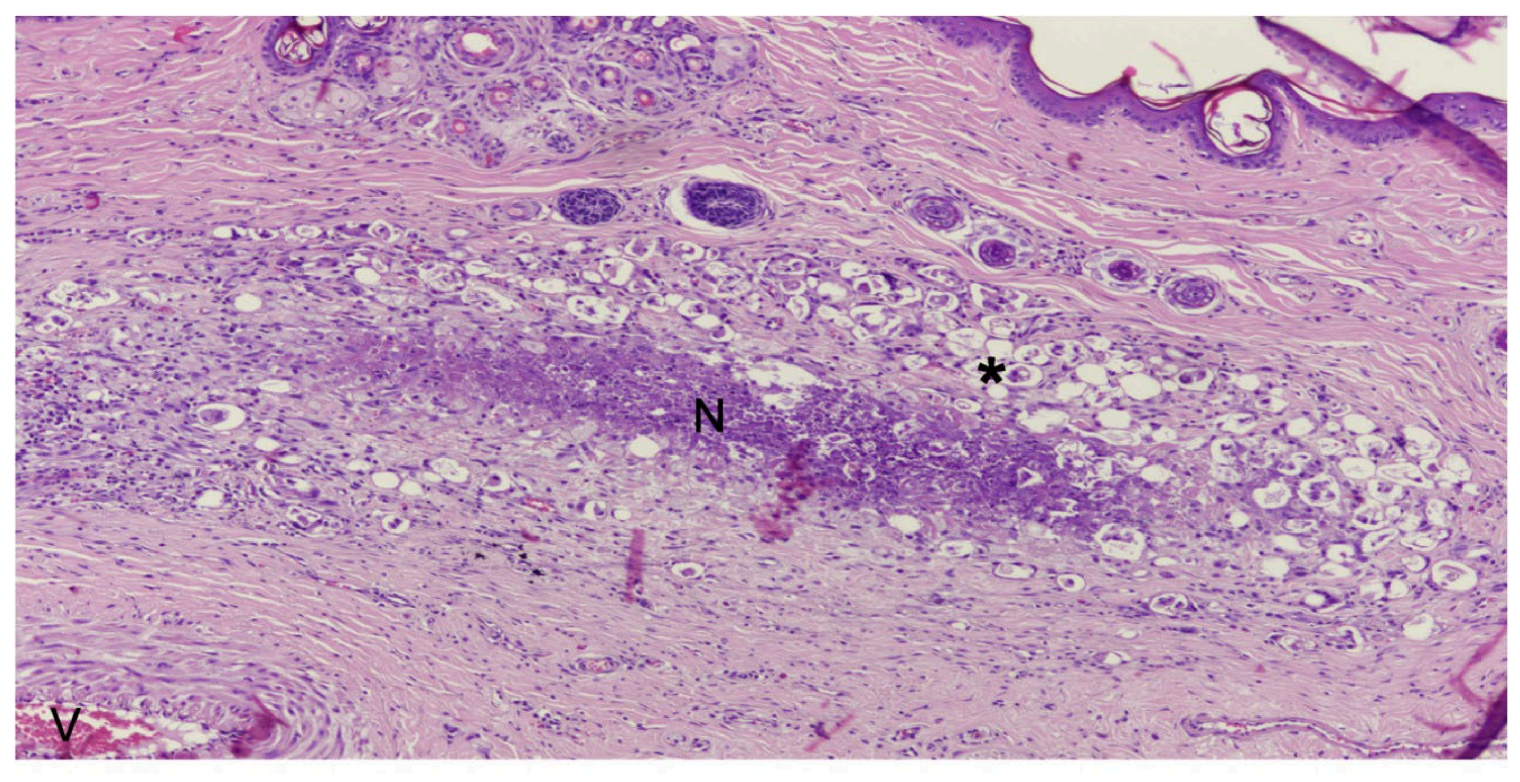
Representative focal necrotic tissue (N) and foreign body reaction (*) adjacent to a large vessel (V) in the distal rabbit ear segment following intra-arterial injection of 0.1 mL Juvelook Volume® at the standard concentration (1:0; 6 mL) (H&E staining).

Similarly, Juvelook Volume®-treated (VAIM Co., Ltd.) ears demonstrated generally preserved vascular architecture. Findings from the proximal segments following 0.1 mL and 0.2 mL injections of the standard preparation and serial dilution groups are shown in Figure 19, with corresponding findings from the middle and distal segments shown in Figure 20 and Figure 21, respectively. Mild foreign body reactions characterised by macrophage infiltration and multinucleated giant cells were occasionally observed adjacent to large vessels within the proximal and middle segments. Focal necrotic changes accompanied by foreign body reactions were occasionally observed in the perivascular tissue of the distal segment; however, these findings did not involve the vascular lumen (Figure 22). No intravascular thrombosis or embolisation was identified.

Overall, histopathological examination demonstrated localised tissue responses consistent with the expected reaction to injectable biomaterials while preserving vascular integrity. No evidence of sustained intravascular obstruction, thrombosis, or embolisation was identified.

## 4. Discussion

The present study demonstrated that intra-arterial injection of Juvelook^®^ and Juvelook Volume^®^ (VAIM Co., Ltd.) did not result in persistent vascular occlusion, skin necrosis, or intravascular thrombosis in the rabbit ear model. Although transient vascular occlusion occurred following the higher injection volume (0.2 mL), particularly in the standard concentration (6 mL) and 12 mL dilution groups, arterial perfusion was consistently restored within 5 min, and no clinically detectable ischaemic injury developed during the 7-day observation period. Histopathological examination confirmed preservation of vascular integrity without sustained luminal obstruction or embolisation. Collectively, these findings demonstrate that injection volume and formulation dilution influence short-term vascular dynamics without resulting in sustained vascular compromise under the experimental conditions investigated.

One of the principal findings of this study was the influence of dilution on vascular behaviour. No transient vascular occlusion was observed following progressive dilution beyond 12 mL, with more uniform intravascular dispersion observed under these conditions. Although the present study did not directly evaluate rheological properties, these observations suggest that particulate concentration influences arterial flow dynamics following intra-arterial injection. Previous studies have demonstrated that vascular compromise associated with injectable biomaterials is influenced by multiple factors, including injection pressure, bolus volume, particle size, suspension characteristics, rheological properties, and injection technique [11,12,13,14]. The present findings extend these observations by demonstrating that dilution alone may substantially alter the short-term vascular behaviour of PDLLA-HA hybrid biostimulators under controlled experimental conditions.

Countercurrent flow was observed exclusively following injection of Juvelook Volume^®^ (VAIM Co., Ltd.), whereas no countercurrent events were detected in the Juvelook^®^ (VAIM Co., Ltd.) cohort. Juvelook Volume^®^ (VAIM Co., Ltd.) contains larger PDLLA microspheres (40–60 μm) and a substantially greater PDLLA content than Juvelook^®^ (VAIM Co., Ltd.), which may contribute to increased resistance to antegrade arterial flow during higher-volume injections. Although rheological testing was beyond the scope of the present study, these physicochemical differences provide a plausible, although unconfirmed, explanation for the formulation-specific vascular responses observed. More broadly, particle concentration, carrier properties, suspension viscosity, and particle morphology are recognised determinants of injectability and tissue distribution in injectable biomaterials [2,16].

Despite transient interruption of arterial blood flow, arterial perfusion was consistently restored within 5 min, and no clinically detectable skin necrosis developed. These findings may be partly explained by the relatively small PDLLA microsphere size (20–60 μm) suspended within a non-crosslinked HA carrier, which may facilitate intravascular dispersion rather than prolonged luminal obstruction. Furthermore, complete restoration of arterial perfusion within 5 min was consistently associated with preservation of tissue viability, suggesting that the transient vascular disturbances observed in this model were insufficient to induce irreversible ischaemic injury. Although retinal artery occlusion, visual loss, and cerebral ischaemic events following PDLLA or related biostimulator injection have been reported, the available literature largely comprises isolated case reports and small numbers of documented events [10,15,17]. Nevertheless, inadvertent intra-arterial injection must not be considered safe, and detailed anatomical knowledge, low-pressure injection, small aliquots, and prompt recognition of vascular compromise remain essential [1,10,11,12,13,14,18].

Histopathological findings corroborated the macroscopic vascular observations. No intravascular thrombosis, embolisation, or persistent luminal occlusion was identified in any experimental group 7 days after injection. Although focal foreign body reactions characterised by macrophage infiltration and multinucleated giant cells were occasionally observed adjacent to blood vessels, these responses were confined to the perivascular tissue and did not compromise vascular patency. These findings are consistent with the expected host response to biodegradable polymeric biomaterials, in which controlled macrophage-mediated tissue remodelling accompanies PDLLA degradation and collagen deposition rather than pathological vascular injury. Previous experimental studies have demonstrated that PDLLA promotes extracellular matrix synthesis, angiogenesis, adipogenesis, and other regenerative responses through macrophage-mediated tissue remodelling. Recent reviews of collagen-stimulating biomaterials have similarly suggested that controlled foreign body reactions form part of their biological mechanism, although excessive or persistent inflammatory responses may contribute to delayed nodules or granulomatous reactions. Accordingly, the mild focal perivascular reactions observed in the present study are more likely to represent an expected biological response than evidence of vascular injury [4,5,6,7,8,9,18].

Although the present study focused specifically on PDLLA-HA hybrid biostimulators, other injectable collagen-stimulating biomaterials, including poly-L-lactic acid, calcium hydroxylapatite, and polycaprolactone, similarly induce tissue regeneration through controlled host responses. However, differences in polymer composition, degradation kinetics, particle morphology, and carrier systems may influence their injectability, tissue integration, and vascular behaviour. Comparative investigations evaluating these biomaterials under standardised experimental conditions are warranted to improve understanding of how formulation-specific characteristics influence vascular behaviour and may facilitate the development of safer injectable biomaterials [9,18,19,20,21,22].

From a biomaterial perspective, the present findings reinforce the importance of formulation design in determining vascular behaviour. The biological and mechanical behaviour of injectable biomaterials depends not only on chemical composition but also on particle size, particle concentration, carrier characteristics, suspension viscosity, and injectability. Recent biomaterials research has emphasised that these physicochemical properties influence tissue distribution, injectability, and translational performance, highlighting the importance of considering biomaterial design alongside injection technique when evaluating vascular safety [21,22].

This study possesses several strengths. To our knowledge, it represents one of the few controlled preclinical investigations directly evaluating the intra-arterial behaviour of commercially available PDLLA-HA hybrid biostimulators under standardised experimental conditions. Both Juvelook^®^ and Juvelook Volume^®^ (VAIM Co., Ltd.) were evaluated using identical protocols, allowing comparison between two formulations differing in particle size and PDLLA content. Furthermore, the paired experimental design reduced inter-animal variability and enabled direct within-animal comparison of injection volumes. The combination of immediate vascular assessment and histopathological evaluation also provided complementary information regarding both acute vascular responses and subsequent tissue changes.

Several limitations should nevertheless be acknowledged. First, Juvelook^®^ and Juvelook Volume^®^ (VAIM Co., Ltd.) were evaluated in separate cohorts rather than in a randomised head-to-head experiment, although identical protocols were applied throughout the study. Second, the relatively small sample size within each experimental group may have limited the ability to detect infrequent vascular events. Third, the rabbit ear model does not fully reproduce the anatomical complexity, vessel calibre, collateral circulation, or haemodynamic characteristics of the human facial arterial system. Fourth, vascular assessment relied primarily on direct visual observation rather than Doppler ultrasonography, angiography, laser speckle contrast imaging, or micro-computed tomography. Fifth, although the injection rate was controlled at approximately 0.2 mL/min, injection pressure was not directly measured, limiting assessment of its contribution to the observed vascular responses. Sixth, outcome assessors were not blinded to treatment allocation, which may have introduced observer bias during the macroscopic and histopathological evaluations. Finally, the observation period was limited to seven days and therefore did not permit assessment of delayed inflammatory or thrombotic events, longer-term vascular changes, or tissue responses associated with PDLLA biodegradation. Accordingly, these findings should be interpreted as reflecting short-term vascular and tissue responses under the experimental conditions investigated. Future studies incorporating direct injection-pressure measurements, objective quantitative vascular imaging, longer follow-up periods, and larger translational animal models are warranted [22].

Taken together, these findings suggest that both biomaterial formulation and injection technique contribute to vascular behaviour following inadvertent intra-arterial injection, emphasising the need to consider both factors when evaluating the safety of injectable biostimulators. Despite the limitations of this experimental model, the present study provides evidence that injection volume and formulation dilution influence the short-term vascular behaviour of PDLLA-HA hybrid biostimulators. These findings contribute to the growing preclinical evidence supporting the vascular evaluation of injectable PDLLA-based biomaterials and provide a foundation for future mechanistic and translational investigations.

## 5. Conclusions

In this rabbit ear intra-arterial model, Juvelook^®^ and Juvelook Volume^®^ (VAIM Co., Ltd.) did not produce persistent vascular occlusion, intravascular thrombosis, embolisation, or skin necrosis following injection volumes of 0.1 mL or 0.2 mL. Transient vascular flow disturbances were observed at higher injection volumes and lower dilution ratios, whereas countercurrent flow occurred only with Juvelook Volume^®^ (VAIM Co., Ltd.). These findings indicate that injection volume and formulation dilution influence the short-term intravascular behaviour of PDLLA-HA hybrid biostimulators and provide preclinical evidence regarding the vascular behaviour of PDLLA-HA hybrid biostimulators under the experimental conditions investigated.

## Figures and Tables

**Table 1:** Experimental groups, dilution ratios, and corresponding final reconstitution volumes of PDLLA-HA biostimulators.

Group	Dilution Ratio	Final Reconstitution Volume (mL)
G1	1:0 (standard preparation)	6
G2	1:1	12
G3	1:2	18
G4	1:3	24
G5	1:4	30
G6	1:5	36
G7	1:6	42

**Table 2:** Diameter of the largest blood vessel in the distal segment following intra-arterial injection of 0.9% NaCl.

Injection Volume (mL)	Mean Diameter (µm)	SD
0.1	56.30	40.484
0.2	162.41	45.551

**Table 3:** Diameter of the largest blood vessel in the distal segment following intra-arterial injection of Juvelook^®^ and Juvelook Volume^®^ at different injection volumes and dilution ratios (n = 7/group).

Volume/Group	1:0 (Undiluted)	1:1	1:2	1:3	1:4	1:5	1:6
Juvelook^®^ (0.1 mL)	138.85 µm	150.13 µm	101.98 µm	151.39 µm	172.81 µm	130.14 µm	143.54 µm
Juvelook^®^ (0.2 mL)	114.22 µm	116.16 µm	168.03 µm	116.10 µm	150.20 µm	139.68 µm	144.04 µm
Juvelook Volume^®^ (0.1 mL)	124.35 µm	144.72 µm	110.35 µm	110.08 µm	144.57 µm	127.86 µm	112.26 µm
Juvelook Volume^®^ (0.2 mL)	145.70 µm	141.19 µm	122.29 µm	138.87 µm	146.87 µm	147.80 µm	137.76 µm

## Data Availability

The data presented in this study are available from the corresponding author upon reasonable request.
